# Novel quinoline/thiazinan-4-one hybrids; design, synthesis, and molecular docking studies as potential anti-bacterial candidates against MRSA†

**DOI:** 10.1039/d3ra01721d

**Published:** 2023-05-12

**Authors:** Asmaa H. Mohamed, Sara M. Mostafa, Ashraf A. Aly, Alaa A. Hassan, Esraa M. Osman, AbdElAziz A. Nayl, Alan B. Brown, Elshimaa M. N. Abdelhafez

**Affiliations:** a Chemistry Department, Faculty of Science, Minia University El-Minia 61519 Egypt asmaa.hamouda@mu.edu.eg sara.ahmed@mu.edu.eg ashrafaly63@yahoo.com ashraf.shehata@mu.edu.eg alaahassan2001@mu.edu.eg esraamah33@gmail.com; b Department of Chemistry, College of Science, Jouf University Sakaka 72341 Aljouf Saudi Arabia aanayel@ju.edu.sa; c Chemistry Department, Florida Institute of Technology Melbourne FL USA; d Department of Medicinal Chemistry, Faculty of Pharmacy, Minia University 61519 Minia Egypt

## Abstract

In an attempt to develop effective and safe antibacterial agents, we synthesized novel thiazinanones by combining the quinolone scaffold and the 1,3-thiazinan-4-one group by reaction between ((4-hydroxy-2-oxo-1,2-dihydroquinolin-3-yl)methylene)hydrazinecarbothioamides and 2,3-diphenylcycloprop-2-enone in refluxing ethanol in the presence of triethyl amine as a catalyst. The structure of the synthesized compounds was characterized by spectral data and elemental analysis, IR, MS, ^1^H and ^13^C NMR spectroscopy which showed two doublet signals for CH-5 and CH-6 and four sharp singlets for the protons of thiazinane NH, CH=N, quinolone NH and OH, respectively. Also, the ^13^C NMR spectrum clearly showed the presence of two quaternary carbon atoms which were assigned to thiazinanone-C-5 and C-6. All the 1,3-thiazinan-4-one/quinolone hybrids were screened for antibacterial activity. Compounds 7a, 7e and 7g showed broad spectrum antibacterial activity against most of the tested strains either G +ve or G −ve. Compound 7e is the most potent antibacterial agent against MRSA with the minimum inhibitory concentration against MRSA found to be 48 μg mL^−1^ compared to the drug ciprofloxacin (96 μg mL^−1^). Additionally, a molecular docking study was performed to understand the molecular interaction and binding mode of the compounds on the active site of *S. aureus Murb* protein. *In silico* docking assisted data strongly correlated with the experimental approach of antibacterial activity against MRSA.

## Abstract 2

In an attempt to develop effective and safe antibacterial agents, thiazinanones bearing the quinolone scaffold were prepared by reaction of the corresponding (quinolinylmethylene)hydrazinecarbothioamides with 2,3-diphenylcycloprop-2-enone.

## Graphical abstract

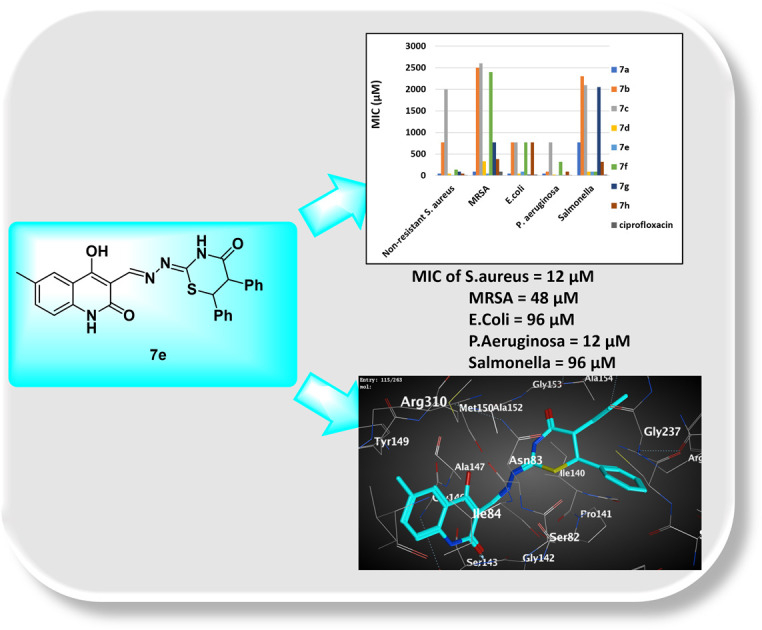

## 1. Introduction

**Fig. 1:**
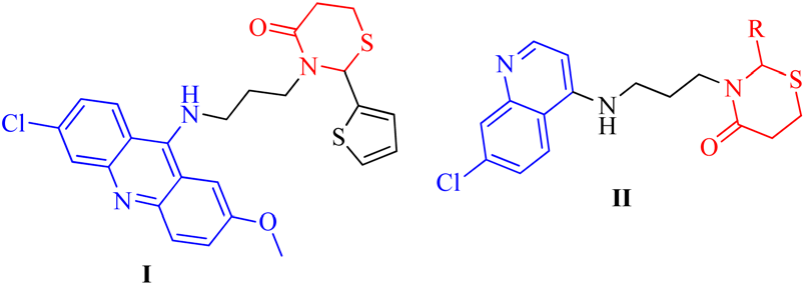
Anticancer and antibacterial thiazinanones I and II.

Thiazinanones, despite their an appropriate term, are very interesting because of their significant role in pharmaceutical chemistry.^1–3^ Substituted thiazinanones displayed antitumor,^4^ antifungal^5^ and antimalarial activity which was assessed by Kumawat *et al.*,^6^ as well as anti-oxidant activity.^7^ Thiazinanone derivatives were obtained through a multicomponent condensation or a two-step process involving an amine, mercapto acid, and carbonyl compounds.^5^ 3-Alkyl-2-aryl-1,3-thiazinan-4-ones with methylsulfonyl pharmacophore exhibited inhibition activity against cyclooxygenase-2-[COX-2].^8^ As well, 3-pyridin-2-ylmethyl-1,3-thiazinan-4-ones displayed anti-oxidant activities.^7^ 3-(3-(6-Chloro-2-methoxyacridin-9-ylamino)propyl)-2-(thiophen-2-yl)-1,3-thiazinan-4-one (I) showed activity against various cancer cell types, such as prostate cancer, two lung cancer cell lines, and eight breast cancer cell lines with varying genetic background (Fig. 1).^9^

Quinolones are a fascinating class of heterocycles having a nitrogen atom. They are also essential moieties in medicinal chemistry. Scientists around the world have been interested in quinolones' biological applications.^10,11^ Quinolone derivatives have revealed anti-cancer,^12^ anti-malarial,^13^ anti-inflammatory,^14^ anti-viral,^15^ anti-bacterial and anti-fungal activities.^16^ 3-((7-Chloroquinolin-4-ylamino)methyl)-2-phenyl-1,3-thiazinan-4-one derivatives II (Fig. 1) were screened for their *in vitro* antibacterial activity against a panel of pathogenic bacterial strains, antitubercular activity against *Mycobacterium tuberculosis* H37Rv and also for their *in vitro* antimalarial activity against *Plasmodium falciparum*. Several of the synthesized compounds exhibited excellent antibacterial activity against *C. tetani*. Some of them showed excellent antitubercular and antimalarial activity.^17^

Many chemists have been interested in the chemistry of cyclopropenones throughout the last three decades,^18,19^ with a special emphasis on diphenylcyclopropenone's behavior.^20^ The formation of aza-cyclopentanones (pyrrolidinones) has been reported, *via* the reaction of 2,3-diphenylcyclopropenone with compounds containing C=N moieties.^21–24^ Triphenylpyrimidinones were obtained through the reaction of amidrazones with 2,3-diphenylcyclopropenone in EtOH/Et_3_N accompanied by elimination of ammonia.^25^ The reaction of alkenylidenehydrazine-carbothioamides with cyclopropenone, as well as the presence of nucleophilic sites like azomethine carbon and sulfur atoms, resulted in 3,5-disubstituted 1,3,4-thiadiazolyl-2,3-diphenylpropenones.^26^ The reaction of cyclopropenone with various aldehyde 4-phenyl thiosemicarbazones in acetic acid afforded pyrrolo[2,1-*b*]oxadiazoles through [2 + 3]cycloaddition; H_2_S was eliminated.^27^ Moreover, 2,4-disubstituted thiosemicarbazides reacted with cyclopropenone to afford the corresponding pyridazines.^28^ 2,3-Diphenylcyclopropenone reacted with *N*-imidoyl-thiourease accompanied by elimination of phenylisothiocyanate; 3-substituted 2,5,6-triphenylpyrimidin-4-ones were obtained.^29^ The reaction of pyrazolylthiourea with cyclopropenone, followed by oxidation with DDQ, yielded 5,6-diphenyl-1,3-thiazinones *via* the formation of pyrazolylimino-3,5,6-triphenyl-1,3-thiazinan-4-ones.^30^ Racemic 2-((2,4-dinitrophenyl)-hydrazono)-5,6-diphenyl-1,3-thiazinan-4-ones and (*Z*)-*N*′-(2,4-dinitrophenyl)-2,3-diphenylacrylo-hydrazides were obtained *via* the diastereoselective reaction between 2,3-diphenylcyclopropenone and 4-substituted 1-(2,4-dinitrophenyl) thiosemicarbazides.^31^

The serious medical problem of Multi Drug Resistance (MDR) of bacteria leads to increasing levels of resistance to classical antibiotics among Gram-positive organisms such as *pneumococci*, *enterococci*, and *staphylococci*.^32^ In communities worldwide, MRSA (methicillin-resistant *Staphylococcus aureus*) is a severe health hazard. The World Health Organization (WHO) has identified MRSA as one of the top threats to people causing developed resistance to almost all classes of antimicrobial agents.^33^ Treatments for MRSA infections are limited and thus it has become a leading cause of morbidity and mortality across the globe after cancer.^34^ In spite of enormous amounts of research works, these MDR pathogens remain a challenge in developing new drug candidates.

**Fig. 2:**
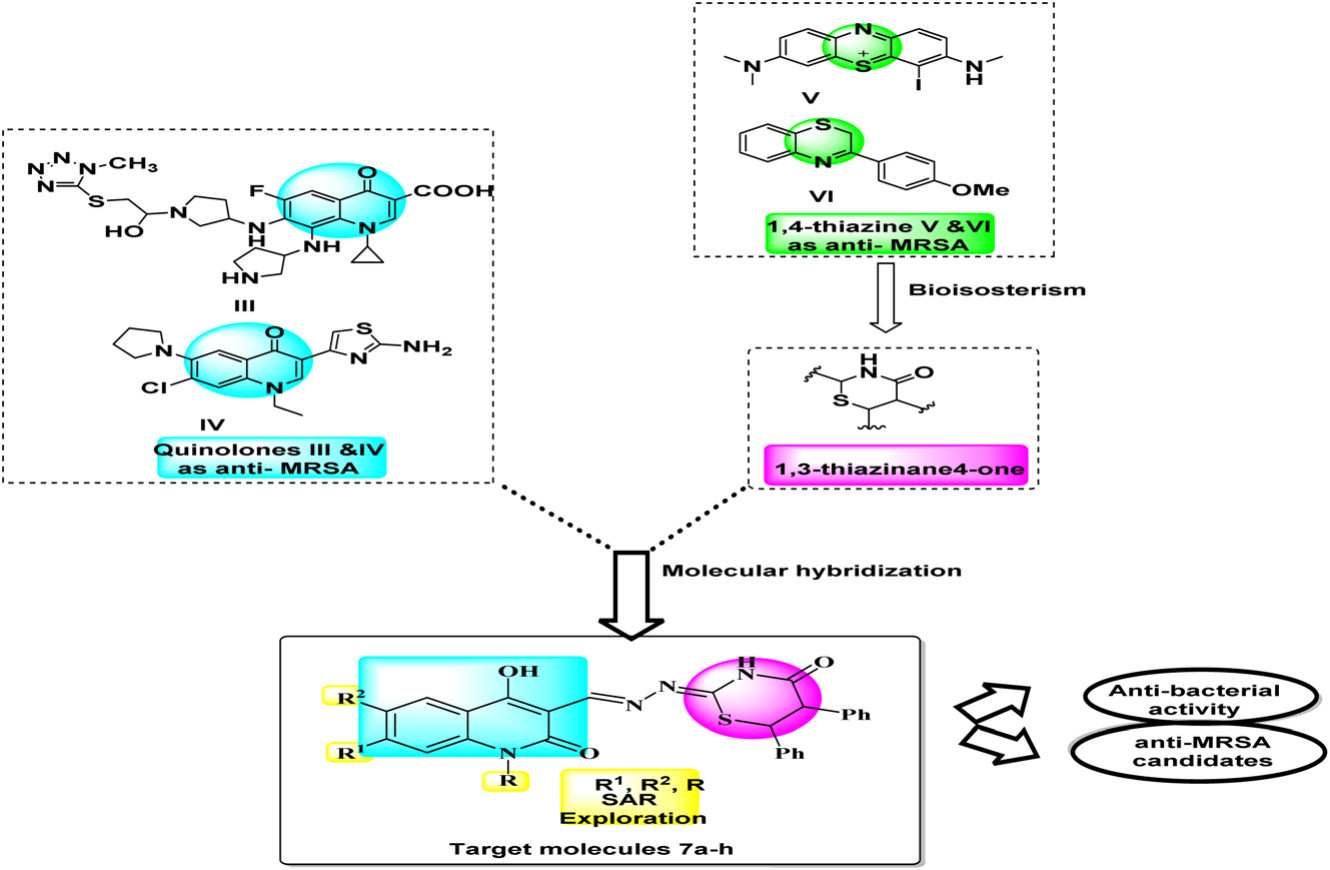
Design of target compounds 7a–h.

Large amounts of effort towards further research of quinolones are performed to develop new more effective antibacterial agents with broader antimicrobial spectrum and better therapeutic index. The azolylthioether quinolones III (Fig. 2) exhibited good antimicrobial activities that displayed remarkable anti-MRSA and anti-*P. aeruginosa* efficacies with low MIC values of 0.25 μg mL^−1^, even superior to reference drugs. They induced bacterial resistance more slowly than clinical drugs.^35^ Also, compound IV (Fig. 2), 3-aminothiazolquinolones, 3-(2-aminothiazol-4-yl)-7-chloro-6-(pyrrolidin-1-yl)quinolone exhibited potent antibacterial activity, low cytotoxicity to hepatocyte cells, strong inhibitory potency to DNA gyrase and a broad antimicrobial spectrum including against multidrug-resistant strains. This active molecule IV also induced bacterial resistance more slowly than norfloxacin.^36^ Moreover, thiazinane was taken into consideration in MDR challenge whereas the monoiodinated thiazine derivative V (Fig. 2) showed good antibacterial activity against methicillin-sensitive *Staphylococcus aureus* (*S. aureus*, MSSA) ATCC 29213 and methicillin-resistant *Staphylococcus aureus* (MRSA) ATCC 43300. Among strategies by which resistance can be achieved, overexpression of efflux pumps such as NorA of *Staphylococcus aureus* leads to a sub-lethal concentration of 3-phenyl-1,4-benzothiazine VI (Fig. 2), at the active site that in turn may predispose the organism to the development of high-level target-based resistance. With an aim to improve both the chemical stability and potency of our previously reported 3-phenyl-1,4-benzothiazine.^37^

In response to the previously mentioned findings, we here designed novel compounds based on the concept of merging more than one scaffold in one compact structure. Hybrids 7a–h gather two types of anti-MRSA scaffolds; 2-quinolones and 1,3-thiazines in one novel hybrid aims to develop simpler and more efficient antibacterial compounds with synergistic effect and less bacterial resistance. Testing against G +ve and G −ve bacteria align with examining against methicillin-resistant *S. aureus* (MRSA) to investigate the anti-MDR activity as well as anti-bacterial spectrum. This is illustrated in a summarized schematic diagram (Fig. 2).

## 2. Results and discussion

### 2.1. Chemistry

**Scheme 1:**
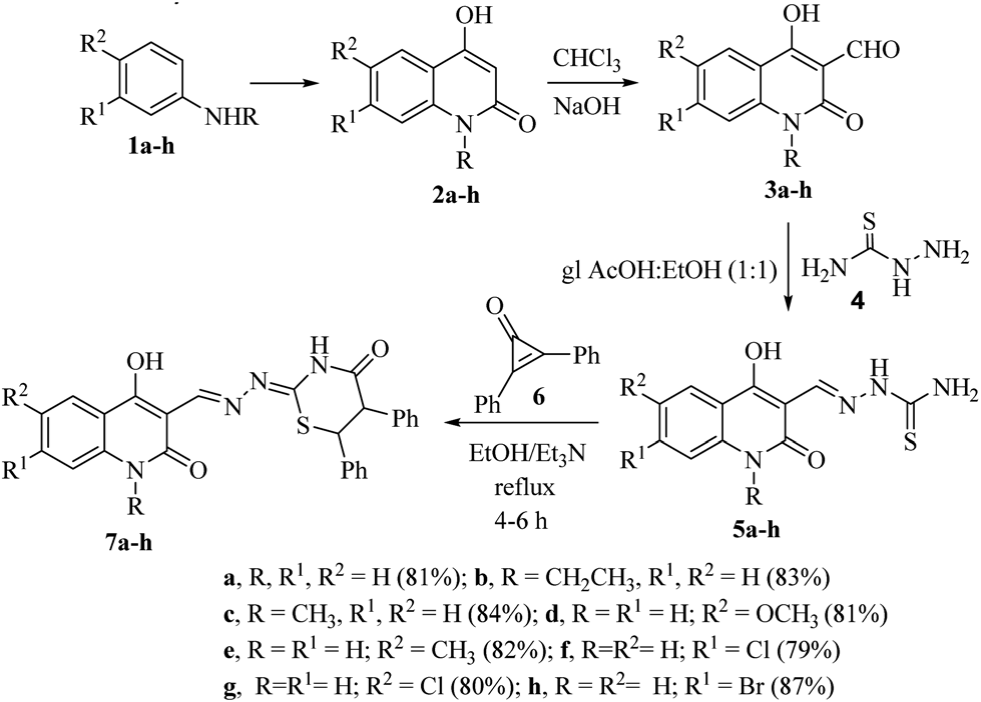
Synthesis of hydrazono-5,6-diphenyl-1,3-thiazinan-4-ones 7a–h.

The target ((*E*)-((4-hydroxy-2-oxo-1,2-dihydroquinolin-3-yl)methylene)hydrazono)-5,6-diphenyl-1,3-thiazinan-4-one derivatives 7a–h were obtained through the route outlined in Scheme 1. The strategy starts by preparing compounds 2a–h, 3a–h and 5a–h according to reported methods. Treating aniline derivatives 1a–h with polyphosphoric acid (PPA), and diethyl malonate (DEM) at 220 °C afforded 2-quinolones 2a–h.^38^ Gentle heating of 2a–h with CHCl_3_ and 15% NaOH gave the corresponding 4-hydroxy-2-oxo-1,2-dihydroquinoline-3-carbaldehydes 3a–h. Reaction of 3a–h with thiosemicarbazide (4) gave the corresponding thiosemicarbazones 5a–h (Scheme 1), and their structures were confirmed by comparing their spectral data to those previously published. Accordingly, thiosemicarbazones 5a–h were treated with 2,3-diphenylcyclopropenone (6) in dry EtOH using a few drops of Et_3_N under reflux for 4–6 h to give 1,3-thiazinan-4-ones 7a–h as the only products in excellent yields.

Diastereomeric mixtures 7a–h were formed as a result of the development of two new stereo centers at C-5 and C-6 positions. As a result, most signals in the ^1^H and ^13^C NMR spectra were duplicated. The expected diastereomeric forms were not separable by column chromatography. In the ^1^H NMR spectra, the signals of the respective protons of the synthesized compounds were confirmed based on their chemical shifts and multiplicities. The compounds reported in this study have been thoroughly characterized by elemental analysis and mass spectral data. The ^1^H NMR spectrum of 7a showed, in addition to the aromatic protons, two doublet signals for CH-6 and CH-5 at *δ*_H_ = 4.50 ppm and 5.44 ppm with coupling constant *J* = 4.0 Hz and four sharp singlets at *δ*_H_ = 8.15, 8.55, 11.60 and 12.03 ppm related to the protons of thiazinane NH, CH=N, quinolone NH and OH, respectively. As the saturated thiazinanes belong to the cyclohexane confirmation structure, the coupling constant values of CH-5 and CH-6 = 4 Hz. Moreover, the ^13^C NMR spectrum clearly showed the presence of two quaternary carbon atom which resonated at *δ*_C_ = 43.36 and 56.06 ppm which were assigned to thiazinanone-CH-6,5. Furthermore, the ^13^C NMR spectrum revealed the presence of carbonyl-thiazinanone and carbonyl-quinolone, quinolone C-4, CH=N and C=N at *δ*_C_ = 168.17, 165.88, 163.30, 161.45 and 158.07 ppm, respectively (see the Experimental section). According to elemental analysis and mass spectrometry, compound 7a has a molecular formula of C_26_H_20_N_4_O_3_S, resulting from the addition of one molecule of hydrazinecarbothioamide 5a with one molecule of 6 without any elimination.

**Fig. 3:**
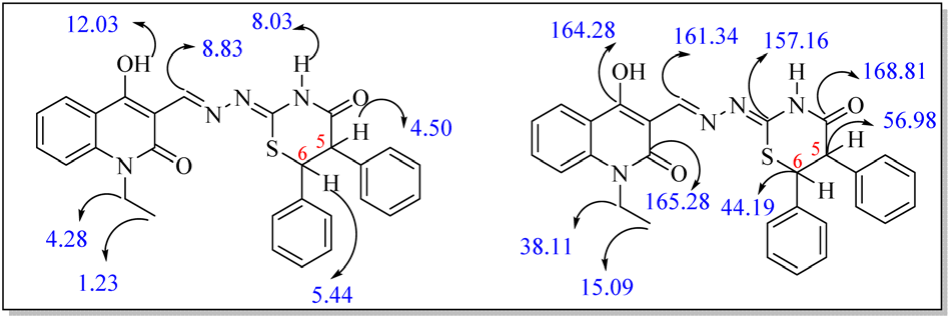
*δ* values of some distinctive carbons and protons of compound 7b.

In case of 7b, its ^1^H NMR spectrum showed triplet and quartet signals for CH_3_ and CH_2_ groups appeared at *δ*_H_ = 1.23 and *δ*_H_ = 4.28 ppm. Whereas the OH, CH=N and thiazinanone-NH protons resonated as three singlets at *δ*_H_ = 12.03, 8.83 and 8.03 ppm, respectively. Also, doublet signals for CH-5 and CH-6 at *δ*_H_ = 4.50 ppm and *δ*_H_ = 5.44 ppm (*J* = 4.0 Hz). The ^13^C NMR spectrum revealed CH_3_, CH_2_, C-6, C-5, C=N, CH=N, C–OH, carbonyl-quinolone and carbonyl-thiazinanone at *δ*_C_ = 15.09, 38.11, 44.19, 56.98, 157.16, 161.34, 164.28, 165.28 and 168.81, respectively (Fig. 3).

**Scheme 2:**
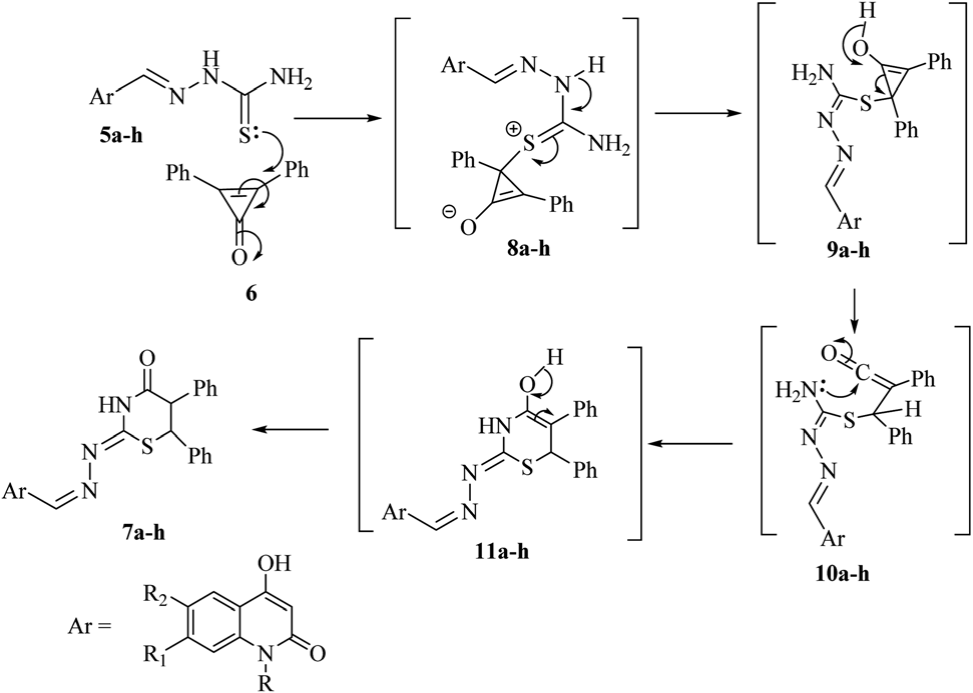
The rationale for the formation of 1,3-thiazinan-4-ones 7a–h.

The plausible mechanism for the formation of 1,3-thiazinan-4-ones 7a–h was based upon the conjugate double bond of 6 was attacked by the thione lone pair forming zwitterion salts 8a–h. Subsequently, a proton was transferred in 8a–h to give the intermediates 9a–h, which on rearrangement and ring opening of cyclopropenone would give the intermediate 10a–h. The carbonyl carbon was then attacked by the lone-pair of nitrogen to form intermediates 11a–h, which rearranged to give the final products 7a–h (Scheme 2).

Using compound 7a as an example, we carried out the reaction in various settings after optimizing the reaction conditions. When the reaction was refluxed in DMF/Et_3_N and dioxane/Et_3_N, it was found that the yield of 7a was reduced to 60% and 64%, respectively. In addition, side products were obtained from the reaction. As a result, utilizing ethanol in the presence of Et_3_N as a catalyst is the best way to get high yields.

### 2.2. Screening of antibacterial activity

The antibacterial and antifungal activities of compounds 7a–h were evaluated *in vitro* against three-Gram positive (G +ve) strains; non-resistant *S. aureus* (ATCC 6538), methicillin resistant *Staphylococcus aureus* (MRSA) and *Escherichia coli* (ATCC 25922) and two-Gram negative (G −ve) strains; *Pseudomonas aeruginosa* (ATCC 10145) and *Salmonella*. The tested compounds were assayed against ciprofloxacin as an antibacterial reference using standard agar cup diffusion method. Results of the antibacterial screening are listed in Table 1.

**Table 1:** The MICs of antibacterial activity of the tested compounds, ciprofloxacin (μg mL^−1^)

Compound	MIC^a^ (μM)				
	Gram +ve			Gram −ve	
	Non-resistant *S. aureus*	MRSA	*E. coli*	*P. aeruginosa*	*Salmonella*
7a	48	96	48	48	768
7b	768	>2000	768	96	>2000
7c	>2000	>2000	768	768	>2000
7d	48	334	48	24	96
7e	12	48	96	12	96
7f	142	>2000	768	320	96
7g	96	768	24	12	>2000
7h	48	384	768	96	320
Ciprofloxacin	12	96	24	12	24

a MIC = lowest conc. inhibit the growth + highest conc. allow the growth/2.

According to the MICs recorded in Table 1, it can be deduced that most of the tested compounds showed a higher antibacterial activity than the reference ciprofloxacin against G +ve bacteria. It was found that compound 7a, 7e and 7h displayed potent activity against non-resistant *S. aureus* compared with the reference with MICs of 12, 48, and 48 μM, respectively. Meanwhile, compounds 7e displayed significant antibacterial activity against MRSA better than the reference with MICs 48 and 96 μM, respectively, however 7a showed remarkable activity of MIC 96 μM.

Moreover, compounds 7g exhibited the good activity against *E. coli* with MICs of 24 μM when compared to the reference, however, both 7a and 7d showed moderate activity against *E. coli*.

**Fig. 4:**
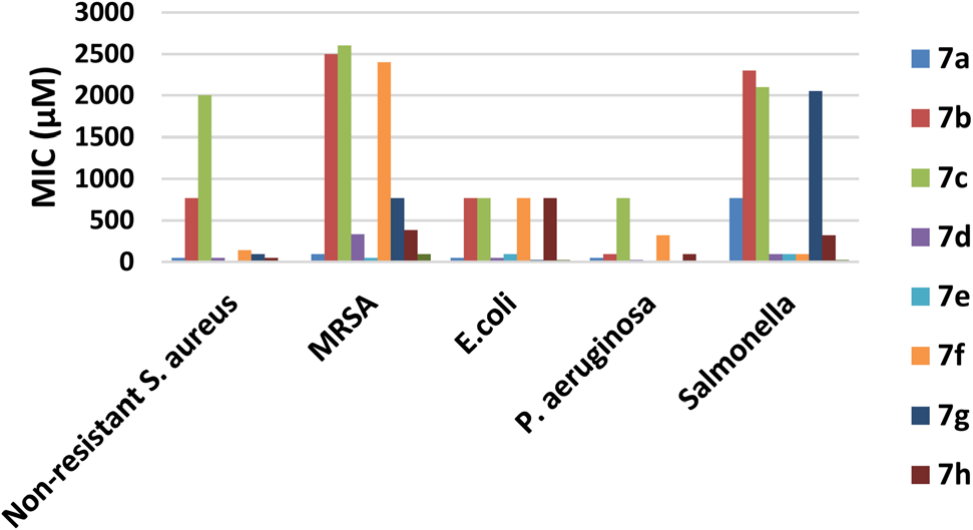
MICs of the tested compounds 7a–h.

Concerning activity against G −ve strains; compounds 7e and 7g revealed a high potency against *P. aeruginosa* with MICs of 12 and 12 μM. On contrast, other compounds displayed moderate to weak activity (Fig. 4). Furthermore, the derivatives 7d–f showed moderate activity against *Salmonella* with MICs of 96 μg mL^−1^, respectively (Table 1 and Fig. 4).

#### 2.2.1. Structure–activity relationship

Based on the aforementioned results, it is obvious that compounds 7a, 7e and 7g showed broad spectrum antibacterial activity against all the tested strains either G +ve or G −ve. In general, the quinolone-based thiazine derivatives 7c and 7f exhibited weak activity almost against most of the tested strains. From the above results, it can be concluded that, there is no specific substituent on the *quinolone nucleus* of tested compounds to enhance the antibacterial activity in a broad-spectrum manner. So, the enhanced activity of some of the tested derivatives may be due to improvement of the physicochemical properties and consequently enhancing permeability to microbial cells.

In summary, compound 7e presented a significant broad spectrum anti-bacterial activity that was probably attributed to when (R^1^ = CH_3_), it would enhance the physicochemical parameters and hence increase cell permeability against either nonresistant or resistant strains.

### 2.3. Molecular modeling studies

Docking studies have been carried out to elucidate the binding mode of the quinolone/thiazine hybrids 7a–h with the protein active site of *S. aureus Murb* (PDB ID: IHSK). Prior to the molecular docking studies, the receptor protein was prepared for docking by omitting additional water and co-factors, followed by the addition of polar hydrogens and computing charges fixation. Also, the docking scores of the tested compounds are depicted in Table 2 that used to calculate the inhibition constant (*K*_i_ value) according to the reported equation^7^ (see ESI†). Typically, a high potency is implied by a low *K*_i_ value and it has to be in the micromolar range for a molecule to be qualified as a lead compound or hit. Compounds 7b, 7c, 7e and 7h have the least *K*_i_ value of 0.62 × 10^−6^, 0.83 × 10^−6^, 0.78 × 10^−6^ and 0.76 × 10^−6^ μM, respectively to qualify as a drug and hence, the most potent among the other tested compounds.

**Table 2:** Energy scores for the complexes formed by the optimized structures of tested 7a–h in the active site of the *S. aureus Murb* (PDB ID: IHSK)

Compound	*S* score	C-Docker energy (kcal mol^−1^)	Inhibition constant, *K*_i_ (μM)	Ligand–receptor interaction		
				Residue	Type	Length (Å)
7a	−7.43	−0.7	3.64 × 10^−6^	SER82	H-donor	3.68
		−0.8		ARG225	Pi-cation	4.32
7b	−8.48	−2.3	0.62 × 10^−6^	SER82	H-donor	2.86
		−2.2		GLY79	H-acceptor	3.00
		−1.4		GLY81	H-acceptor	3.24
		−0.0		TYR149	Pi–Pi	3.94
7c	−8.27	−2.1	0.83 × 10^−6^	SER82	H-donor	2.94
		−2.2		GLY79	H-acceptor	3.00
		−1.2		GLY81	H-acceptor	3.26
		−0.0		TYR149	Pi–Pi	3.94
7d	−8.00	−2.1	1.39 × 10^−6^	GLY146	H-donor	3.19
		−2.2		SER82	H-acceptor	3.12
		−1.6		SER82	H-acceptor	2.60
		−0.7		TYR149	H–Pi	4.10
		−2.6		ASN83	Pi–H	4.15
7e	−8.34	−1.7	0.78 × 10^−6^	AS80	H-acceptor	3.30
		−1.9		SER143	H-acceptor	3.28
		−0.7		SER82	Pi–H	4.55
		−0.9		ARG225	Pi-cation	4.36
7f	−7.95	−2.0	1.51 × 10^−6^	SER82	H-donor	2.99
		−2.1		GLY79	H-acceptor	2.99
		−0.9		GLY81	H-acceptor	3.32
		−0.6		ILE140	Pi–H	4.51
		−0.6		ILE140	Pi–H	4.10
		−0.0		TRY149	Pi–Pi	3.93
7g	−7.69	−0.9	2.34 × 10^−6^	SER82	H-acceptor	3.99
		−1.5		SER82	H-acceptor	2.86
		−0.7		TRY149	Pi–H	4.08
7h	−8.36	−2.4	0.76 × 10^−6^	ASN80	H-acceptor	3.14
		−1.0		SER143	H-acceptor	3.49
		−0.7		SER80	Pi–H	4.52
		−0.9		ARG225	Pi-cation	4.36
Ciprofloxacin	−6.53	−1.5	14.49 × 10^−6^	SER82	H-acceptor	3.19
		−2.7		GLY79	H-acceptor	2.74

**Fig. 5:**
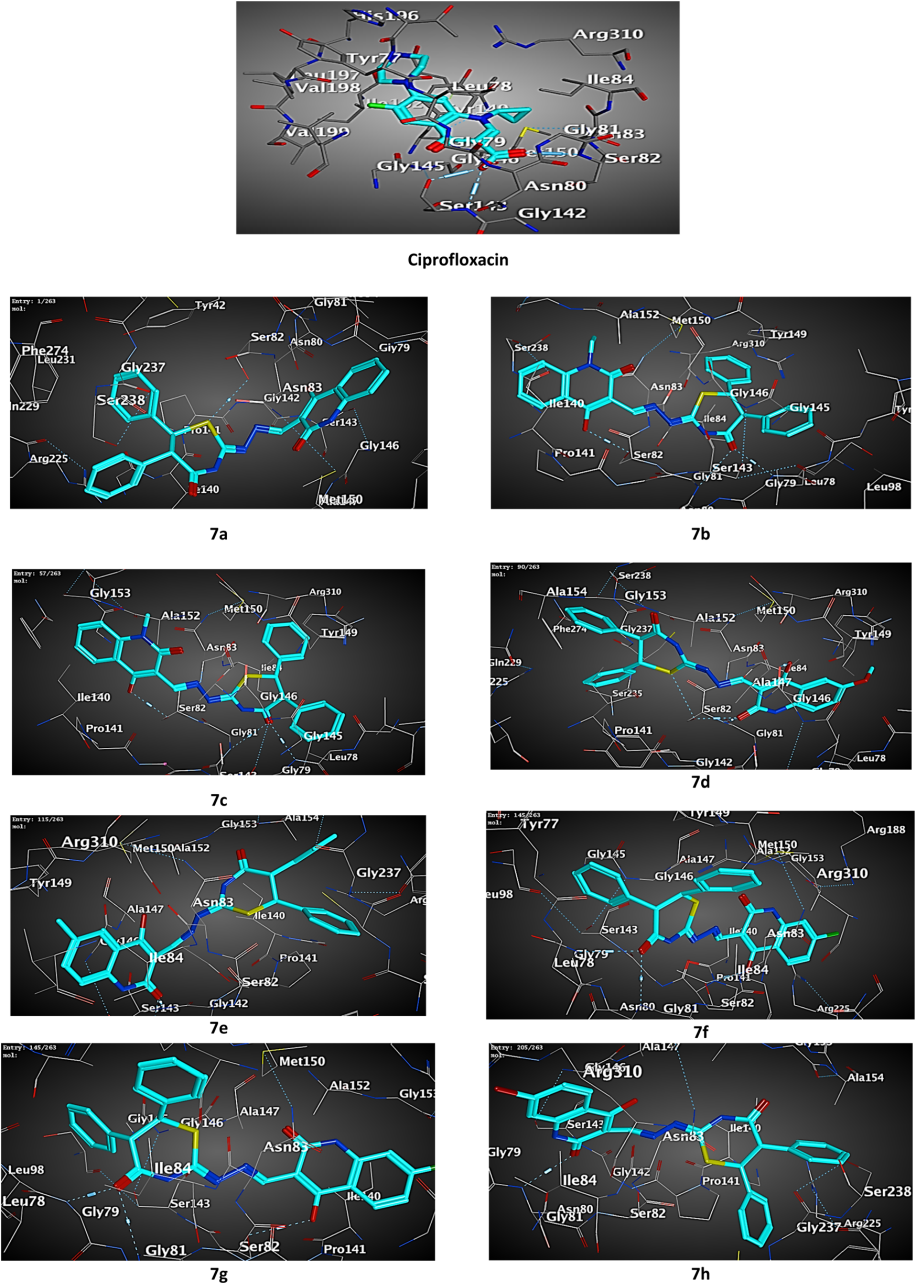
3D diagram illustrating the binding modes of the optimized structures of tested 7a–h in the active site of the *S. aureus Murb* (PDB ID: IHSK).

Docking results of the known antibacterial reference; ciprofloxacin into active site of *S. aureus Murb* protein (Fig. 5 and Table 2) revealed that ciprofloxacin showed CDOCKER energy of −6.53 kcal mol^−1^ and engaged in two hydrogen bonds with amino acid residues SER82 and GLY79.

Most of the tested compounds have high binding affinity to protein of *S. aureus Murb* as the binding free energy (Δ*G*) values of them range from 0.0 to −2.6 kcal mol^−1^. The docking study results of target 7b, 7c and 7f showed interactions typically as the reference with amino acid residues SER82 and GLY79.

Although, all the tested compounds 7a–h showed interaction with amino acid residues SER82 and TYR149, hybrids 7a, 7e and 7h lack interaction with the last residue.

Moreover, compounds 7e and 7h exhibited potential interactions with both amino acid residues ASN80 and ARG255, while the hybrid 7d interacted with the first residue and the second residue engaged with the 7a.

Interestingly, compound 7f showed additional two hydrophobic interactions with ILE140 amino acid residue which is not observed with the others.

Collectively, the docking results were in agreement with the biological study, and we could conclude that hybrid 7e entitled to be promising as attractive future lead candidate for the development of broad-spectrum antibacterial activity.

## 3. Experimental

### 3.1. Chemistry

A list of chemicals and instrumentation is provided in the ESI.†

#### 3.1.1. Starting materials

Carbaldehydes 3a–c,^39–41^3e–h^42^ and thiosemicarbazones 5a–c^43,44^ and 5e–h^45^ were prepared according to literature methods.

#### 3.1.2. General procedure

Equimolar amounts of 2,3-diphenylcycloprop-2-enone 6 and the appropriate hydrazinecarbothioamides 5a–e were mixed in absolute EtOH and a few drops of Et_3_N was added as a catalyst and refluxed for about 4–6 h, furnished yellow precipitates (*i.e.* the reaction was followed up by TLC analysis). The precipitate was filtered, washed with ethanol, dried and recrystallized from the stated solvents to give the final products 7a–h.

##### (*Z*)-2-((*E*)-((4-Hydroxy-2-oxo-1,2-dihydroquinolin-3-yl)methylene)-hydrazono)-5,6-diphenyl-1,3-thiazinan-4-one (7a)

Yellow crystals (DMF/EtOH), yield: 0.379 g (81%); mp 300–302 °C; *R*_f_ = 0.22 (toluene–ethyl acetate, 1 : 1); IR (KBr): *ν* = 3392 (OH), 3230, 3215 (NH), 3066 (Ar-CH), 2934 (aliph-CH), 1662 and 1649 (CO), 1628 cm^−1^ (C=N); ^1^H NMR (400 MHz, DMSO-*d*_6_): *δ*_H_ = 4.50 (d, 1H, *J* = 4.0 Hz, thiazinanone-H6), 5.44 (d, 1H, *J* = 4.0 Hz, thiazinanone-H5), 6.84 (dd, 2H, *J* = 8.0 Hz, Ar-H), 6.96 (dd, 2H, *J* = 8.0 Hz, Ar-H), 7.18–7.30 (m, 5H, Ar-H), 7.58–7.60 (m, 5H, Ar-H), 8.15 (brs, 1H, thiazinanone-NH), 8.55 (s, 1H, CH=N), 11.60 (s, 1H, quinolone-NH), 12.03 (s, 1H, quinolone-OH); ^13^C NMR (100 MHz, DMSO-*d*_6_): *δ*_C_ = 43.36 (thiazinanone-CH-6), 56.06 (thiazinanone-CH-5), 109.90 (quinolone-C3), 114.87, 116.49, 117.75, 118.41, 120.03, 122.02, 122.68, 123.27, 125.56, 126.59, 127.55, 128.30, 129.83, 132.05 (Ar-CH), 134.99, 136.98, 139.57, 143.77 (Ar-C), 158.07 (C=N), 161.45 (CH=N), 163.30 (C-OH), 165.88 (quinolone-C=O), 168.17 (thiazinanone-C=O). MS (Fab, 70 eV, %): *m*/*z* = 468 (M^+^, 70), 391 (25), 307 (100), 289 (15), 273 (5). Anal. calcd for C_26_H_20_N_4_O_3_S (468.53): C, 66.65; H, 4.30; N, 11.96; S, 6.84. Found: C, 66.80; H, 4.34; N, 12.10; S, 6.98.

##### (*Z*)-2-((*E*)-((1-Ethyl-4-hydroxy-2-oxo-1,2-dihydroquinolin-3-yl)-methylene)hydrazono)-5,6-diphenyl-1,3-thiazinan-4-one (7b)

Yellow crystals (DMF/EtOH), yield: 0.412 g (83%); mp 281–283 °C; *R*_f_ = 0.30 (toluene–ethyl acetate, 10 : 8); IR (KBr) *ν* = 3390 (OH), 3230 (NH), 3070 (Ar-CH), 2970 (aliph-CH), 1672 and 1668 (CO), 1611 cm^−1^ (C=N); ^1^H NMR (400 MHz, DMSO-*d*_6_): *δ*_H_ = 1.23 (t, 3H, *J* = 8.0 Hz, CH_3_), 4.28 (q, 2H, *J* = 8.0 Hz, CH_2_), 4.50 (d, 1H, *J* = 4.0 Hz, thiazinanone-H6), 5.44 (d, 1H, *J* = 4.0 Hz, thiazinanone-H5), 6.84 (dd, 2H, Ar-H), 6.97 (dd, 2H, Ar-H), 7.28–7.31 (m, 10H, Ar-H), 8.03 (brs, 1H, thiazinanone-NH), 8.83 (s, 1H, CH=N), 12.03 (s, 1H, quinolone-OH); ^13^C NMR (100 MHz, DMSO-*d*_6_): *δ*_C_ = 15.09 (CH_3_), 38.11 (CH_2_), 44.19 (thiazinanone-CH-6), 56.98 (thiazinanone-CH-5), 109.89 (quinolone-C3), 115.30, 123.90, 123.99, 127.94, 128.02, 128.59 (Ar-CH), 128.79, 128.90, 129.24, 129.39 (Ar-2CH), 133.50, 133.63, 134.62, 139.80 (Ar-C), 157.16 (C=N), 161.34 (CH=N), 164.28 (C-OH), 165.28 (quinolone-C=O), 168.81 (thiazinanone-C=O); MS (Fab, 70 eV, %): *m*/*z* = 496 (M^+^, 100), 468 (70), 307 (50), 316 (88), 288 (45), 280 (20), 216 (15), 189 (30). Anal. calcd for C_28_H_24_N_4_O_3_S (496.58): C, 67.72; H, 4.87; N, 11.28; S, 6.46. Found: C, 67.81; H, 4.92; N, 11.15; S, 6.33.

##### (*Z*)-2-((*E*)-((1-Methyl-4-hydroxy-2-oxo-1,2-dihydroquinolin-3-yl)-methylene)hydrazono)-5,6-diphenyl-1,3-thiazinan-4-one (7c)

Yellow crystals (DMF/H_2_O), yield: 0.405 g (84%); mp 280–282 °C; *R*_f_ = 0.22 (toluene–ethyl acetate, 10 : 8); IR (KBr) *ν* = 3308 (OH), 3276 (NH), 3109 (Ar-CH), 2965 (aliph-CH), 1639 and 1615 (CO), 1584 cm^−1^ (C=N); ^1^H NMR (400 MHz, DMSO-*d*_6_): *δ*_H_ = 3.61 (s, 3H, CH_3_), 4.51 (d, 1H, *J* = 4.0 Hz, thiazinanone-H6), 5.45 (d, 1H, *J* = 4.0 Hz, thiazinanone-H5), 6.84–6.86 (m, 2H, Ar-H), 6.97 (dd, 2H, Ar-H), 7.23–7.30 (m, 10H, Ar-H), 7.97 (brs, 1H, thiazinanone-NH), 8.83 (s, 1H, CH=N), 12.03 (s, 1H, quinolone-OH); ^13^C NMR (100 MHz, DMSO-*d*_6_): *δ*_C_ = 34.48 (CH_3_), 44.19 (thiazinanone-CH-6), 55.76 (thiazinanone-CH-5), 108.56 (quinolone-C3), 114.11, 122.27, 122.75, 126.61, 126.93, 128.07 (Ar-CH), 128.40, 128.52, 129.47, 129.73 (Ar-2CH), 133.74, 133.77, 134.81, 141.52 (Ar-C), 157.97 (C=N), 161.29 (CH=N), 164.65 (C-OH), 165.69 (quinolone-C=O), 168.38 (thiazinanone-C=O); MS (Fab, 70 eV, %): *m*/*z* = 482 (M^+^, 70), 468 (50), 316 (80), 307 (100), 286 (45), 280 (90), 202 (10), 175 (30), 161 (50). Anal. calcd for C_27_H_22_N_4_O_3_S (482.55): C, 67.20; H, 4.60; N, 11.61; S, 6.64. Found: C, 67.31; H, 4.64; N, 11.55; S, 6.58.

##### (*Z*)-2-((*E*)-((4-Hydroxy-6-methoxy-2-oxo-1,2-dihydroquinolin-3-yl)methylene)hydrazono)-5,6-diphenyl-1,3-thiazinan-4-one (7d)

Yellow crystals (DMF/H_2_O), yield: 0.403 g (81%); mp 295–297 °C; *R*_f_ = 0.18 (toluene–ethyl acetate, 1 : 1); IR (KBr): *ν* = 3334 (OH), 3276, 3190 (NH), 3110 (Ar-CH), 2865 (aliph-CH), 1661 and 1625 (CO), 1593 cm^−1^ (C=N); ^1^H NMR (400 MHz, DMSO-*d*_6_): *δ*_H_ = 3.77 (s, 3H, OCH_3_), 4.48 (d, 1H, *J* = 4.0 Hz, thiazinanone-H6), 5.45 (d, 1H, *J* = 4.0 Hz, thiazinanone-H5), 6.85–6.86 (m, 2H, Ar-H), 6.95–6.97 (m, 1H, Ar-H), 7.23–7.31 (m, 10H, Ar-H), 7.98 (brs, 1H, thiazinanone-NH), 8.76 (s, 1H, CH=N), 11.48 (s, 1H, quinolone-NH), 12.03 (s, 1H, quinolone-OH); ^13^C NMR (100 MHz, DMSO-*d*_6_): *δ*_C_ = 44.20 (thiazinanone-CH-6), 55.36 (OCH_3_), 56.30 (thiazinanone-CH-5), 109.31 (quinolone-C3), 116.61 (Ar-2CH), 125.26, 126.92, 127.15, 127.24 (Ar-CH), 128.06, 128.31 (Ar-2CH), 128.47 (Ar-CH), 130.09 (Ar-2CH), 132.54, 133.10, 136.90, 138.83, 143.80 (Ar-C), 153.64 (C=N), 156.61 (CH=N), 162.78 (C-OH), 164.81 (quinolone-C=O), 167.09 (thiazinanone-C=O); MS (Fab, 70 eV, %): *m*/*z* = 498 (M^+^, 30), 468 (60), 307 (100), 280 (85), 218 (14), 191 (20). Anal. calcd for C_27_H_22_N_4_O_4_S (498.55): C, 65.05; H, 4.45; N, 11.24; S, 6.43. Found: C, 65.17; H, 4.48; N, 11.17; S, 6.53.

##### (*Z*)-2-((*E*)-((4-Hydroxy-6-methyl-2-oxo-1,2-dihydroquinolin-3-yl)-methylene)hydrazono)-5,6-diphenyl-1,3-thiazinan-4-one (7e)

Yellow crystals (DMF/MeOH), yield: 0.314 g (82%); mp = 304–306 °C; *R*_f_ = 0.20 (toluene–ethyl acetate, 10 : 8); IR (KBr): *ν* = 3410 (OH), 3215, 3211 (NH), 3062 (Ar-CH), 2925 (aliph-CH), 1668 and 1648 (CO), 1620 cm^−1^ (C=N); ^1^H NMR (400 MHz, DMSO-*d*_6_): *δ*_H_ = 2.78 (s, 3H, CH_3_), 4.55 (d, 1H, *J* = 4.0 Hz, thiazinanone-H6), 5.53 (d, 1H, *J* = 4.0 Hz, thiazinanone-H5), 7.01 (dd, 2H, *J* = 8.0 Hz, Ar-H), 7.25–7.54 (m, 10H, Ar-H), 7.89 (s, 1H, Ar-H), 8.10 (brs, 1H, thiazinanone-NH), 8.56 (s, 1H, CH=N), 11.55 (s, 1H, quinolone-NH), 12.56 (s, 1H, quinolone-OH); ^13^C NMR (100 MHz, DMSO-*d*_6_): *δ*_C_ = 20.02 (CH_3_), 51.26 (thiazinanone-CH-6), 54.88 (thiazinanone-CH-5), 109.27, (quinolone-C3), 110.26, 110.35, 114.08, 114.17, 114.94, 115.05, 116.35, 117.17, 118.22, 123.91, 124.93, 125.54, 129.71, (Ar-CH), 131.14, 137.80 (Ar-C), 139.29 (Ar-2C), 140.12 (Ar-C), 160.72 (C=N), 161.45 (CH=N), 164.99 (C-OH), 165.10 (quinolone-C=O), 166.16 (thiazinanone-C=O). MS (Fab, 70 eV, %): *m*/*z* = 482 (M^+^, 58), 391 (26), 316 (78), 309 (100), 286 (45), 282 (35), 202 (10), 175 (28), 161 (36). Anal. calcd for C_27_H_22_N_4_O_3_S (482.55): C, 67.20; H, 4.60; N, 11.61; S, 6.64. Found: C, 67.36; H, 4.64; N, 11.75; S, 6.73.

##### (*Z*)-2-((*E*)-((7-Chloro-4-hydroxy-2-oxo-1,2-dihydroquinolin-3-yl)methylene)hydrazono)-5,6-diphenyl-1,3-thiazinan-4-one (7f)

Yellow crystals (DMF), yield: 0.295 g (79%); mp = 310–312 °C; *R*_f_ = 0.15 (toluene–ethyl acetate, 1 : 1); IR (KBr): *ν* = 3402 (OH), 3221, 3216 (NH), 3042 (Ar-CH), 2920 (Ali-CH), 1670, 1640 (CO), 1588 cm^−1^ (C=N). ^1^H NMR (400 MHz, DMSO-*d*_6_): *δ*_H_ = 4.54 (d, 1H, *J* = 4.0 Hz, thiazinanone-H6), 5.49 (d, 1H, *J* = 4.0 Hz, thiazinanone-H5), 6.81 (dd, 2H, *J* = 8.0 Hz, Ar-H), 7.19–7.58 (m, 10H, Ar-H), 7.90 (s, 1H, Ar-H), 8.12 (brs, 1H, thiazinanone-NH), 8.76 (s, 1H, CH=N), 12.62 (s, 1H, quinolone-NH), 13.21 (s, 1H, quinolone-OH); ^13^C NMR (100 MHz, DMSO-*d*_6_): *δ*_C_ = 45.05 (thiazinanone-CH-6), 56.14 (thiazinanone-CH-5), 100.10 (quinolone-C3), 113.65, 114.91, 115.12, 115.85, 116.20, 122.02, 123.01, 123.27, 125.50 (Ar-CH), 126.05 (Ar-2CH), 128.41, 129.98 (Ar-CH), 131.89, 135.02, 136.98, 138.86, 142.78 (Ar-C), 156.12 (C=N), 161.46 (CH=N), 163.45 (C-OH), 165.35 (quinolone-C=O), 166.24 (thiazinanone-C=O); MS (Fab, 70 eV, %): *m*/*z* = 504 (M + 2, 40), 503 (M + 1, 15), 502 (M^+^, 7), 391 (10), 309 (5), 308 (12), 307 (40), 289 (17), 260 (5), 154 (100). Anal. calcd for C_26_H_19_ClN_4_O_3_S (502.97): C, 62.09; H, 3.81; N, 11.14; S, 6.38. Found: C, 62.24; H, 3.85; N, 11.28; S, 6.48.

##### (*Z*)-2-((*E*)-((6-Chloro-4-hydroxy-2-oxo-1,2-dihydroquinolin-3-yl)methylene)hydrazono)-5,6-diphenyl-1,3-thiazinan-4-one (7g)

Yellow crystals (DMF/EtOH), yield: 0.402 g (80%); mp 270–272 °C; *R*_f_ = 0.18 (toluene–ethyl acetate, 1 : 1); IR (KBr): *ν* = 3330 (OH), 3282, 3178 (NH), 3089 (Ar-CH), 2860 (aliph-CH), 1670 and 1652 (CO), 1627 cm^−1^ (C=N); ^1^H NMR (400 MHz, DMSO-*d*_6_): *δ*_H_ = 4.50 (d, 1H, *J* = 4.0 Hz, thiazinanone-H6), 5.44 (d, 1H, *J* = 4.0 Hz, thiazinanone-H5), 6.85–6.86 (m, 2H, Ar-H), 6.95–6.97 (m, 1H, Ar-H), 7.23–7.30 (m, 10H, Ar-H), 8.03 (brs, 1H, thiazinanone-NH), 8.63 (s, 1H, CH=N), 11.62 (s, 1H, quinolone-NH), 12.03 (s, 1H, quinolone-OH); ^13^C NMR (100 MHz, DMSO-*d*_6_): *δ*_C_ = 44.19 (thiazinanone-CH-6), 56.89 (thiazinanone-CH-5), 109.86 (quinolone-C3), 115.32, 122.10, 123.90, 127.92, 128.01, 128.53, 128.95, 129.24, 129.39 (Ar-CH), 133.54, 134.80 (Ar-2CH), 132.40, 133.30, 133.60, 136.32, 143.76 (Ar-C), 157.26 (C=N), 161.32 (CH=N), 162.57 (C-OH), 164.89 (quinolone-C=O), 168.71 (thiazinanone-C=O); MS (Fab, 70 eV, %): *m*/*z* = 503 (M + 1, 30), 502 (M^+^, 55), 468 (40), 307 (20), 280 (16), 223/222 (7/50), 196/195 (100/64). Anal. calcd for C_26_H_19_ClN_4_O_3_S (502.97): C, 62.09; H, 3.81; N, 11.14; S, 6.38. Found: C, 62.16; H, 3.86; N, 11.26; S, 6.48.

##### (*Z*)-2-((*E*)-((7-Bromo-4-hydroxy-2-oxo-1,2-dihydroquinolin-3-yl)-methylene)hydrazono)-5,6-diphenyl-1,3-thiazinan-4-one (7h)

Yellow crystals (DMF/EtOH), yield: 0.325 g (87%); mp = 338–340 °C; *R*_f_ = 0.20 (toluene–ethyl acetate, 1 : 1); IR (KBr): *ν* = 3390 (OH), 3219, 3215 (NH), 3072 (Ar-CH), 2914 (aliph-CH), 1661 and 1650 (CO), 1600 cm^−1^ (C=N). ^1^H NMR (400 MHz, DMSO-*d*_6_): *δ*_H_ = 4.54 (d, 1H, *J* = 4.0 Hz, thiazinanone-H6), 5.55 (d, 1H, *J* = 4.0 Hz, thiazinanone-H5), 6.99 (dd, 2H, *J* = 8.0 Hz, Ar-H), 7.13–7.33 (m, 10H, Ar-H), 7.90 (m, 1H, Ar-H), 8.12 (brs, 1H, thiazinanone-NH), 8.55 (s, 1H, CH=N), 12.20 (s, 1H, quinolone-NH), 12.91 (s, 1H, quinolone-OH). ^13^C NMR (100 MHz, DMSO-*d*_6_): *δ*_C_ = 46.14 (thiazinanone-CH-6), 55.34 (thiazinanone-CH-5), 109.33 (quinolone-C3), 110.50, 112.35, 114.15, 114.98, 115.22, 116.32, 117.22, 119.54, 122.65, 123.34, 125.30 (Ar-CH), 129.80 (Ar-2CH), 131.14 (Ar-2C), 136.89, 138.40, 141.09 (Ar-C), 160.80 (C=N), 161.14 (CH=N), 164.98 (C-OH), 165.24 (quinolone-C=O), 167.23 (thiazinanone-C=O). MS (70 eV, %): *m*/*z* 549 (M + 2, 25), 547 (M^+^, 25), 530 (17), 529 (14), 476 (15), 460 (100), 443 (25), 391 (30), 375 (22), 330 (38), 305 (40), 154 (100). Anal. calcd for C_26_H_19_BrN_4_O_3_S (547.42): C, 57.05; H, 3.50; N, 10.23; S, 5.86. Found: C, 57.23; H, 3.55; N, 10.38; S, 5.96.

### 3.2. Biology

#### 3.2.1. Screening of antibacterial activity

The antibacterial activity was screened according to serial dilution method. Minimal inhibition concentration (MIC) is the lowest concentration of an antimicrobial agent that can inhibit the visible growth of a microorganism after overnight incubation (see ESI†).

#### 3.2.2. Molecular docking study

The docking simulation study was carried out using Molecular Operating Environment (MOE®) version 2014.09 (Chemical Computing Group Inc., Montreal, QC, Canada). The computational software operated under “Windows XP” installed on an Intel Pentium IV PC with a 1.6 GHz processor and 512 MB memory. The target compounds were constructed into a 3D model using the builder interface of the MOE program and docked into the active site of caspase-3 (PDB: 3GJQ). Checking their structures and the formal charges on atoms by 2D depiction was carried out and the energy, was minimized until an RMSD (root mean square deviations) gradient of 0.01 kcal mol^−1^ and RMS (Root Mean Square) distance of 0.1 A with MMFF94X (Merck molecular force field 94X) force-field and the partial charges were automatically calculated (see ESI†).

## 4. Conclusions

In short, a series of 1,3-thiazinanone derivatives have been synthesized in excellent yields *via* nucleophilic attack of thiosemicarbazones on 2,3-diphenylcyclopropenone. The target compounds were identified and characterized using ^1^H NMR, ^13^C NMR, MS and elemental analysis. The suggested mechanism for the formation of the final products was remembered. The biological results revealed that some target compounds exhibited good antibacterial activity against most of the tested G +ve and G −ve strains, especially compound 7e against MRSA even superior to reference drug. They induced bacterial resistance more slowly than clinical drugs. Molecular docking study indicated strong binding interaction of the tested compounds. In conclusion, compound 7e revealed potential broad spectrum anti-bacterial activity that should be taken into consideration as good candidates for further study.

## Author contributions

A. H. M. (writing, editing, and revision); S. M. M. (revision), A. A. A. (concept, writing, edit, revision, and submitting), A. A. H. (editing), E. M. O. (experimental), A. B. B. (editing and revision), E.-S. M. N. A. (biology, writing, and editing). All authors have read and agreed to the published version of the manuscript.

## Conflicts of interest

The authors declare no conflict of interest. The authors declare that they have no known competing interests.

## Supplementary Material

RA-013-D3RA01721D-s001
