# Supplementary material for: Novel quinoline/thiazinan-4-one hybrids; design, synthesis, and molecular docking studies as potential anti-bacterial candidates against MRSA
Source: RSC Adv. 2023 May 12;13(21):14631–40. doi: 10.1039/d3ra01721d (PMC10178015; doi:10.1039/d3ra01721d)
Supplement: RA-013-D3RA01721D-s001 [file RA-013-D3RA01721D-s001.pdf]

Electronic Supplementary Material (ESI) for RSC Advances.
This journal is © The Royal Society of Chemistry 2023

Supplementary File

Novel Quinoline /Thiazinan-4-One Hybrids; Design, Synthesis, And Molecular Docking Studies as Potential Anti-Bacterial Candidate Against MRSA

Asmaa H. Mohamed,[a] Sara M. Mostafa,[a] Ashraf A. Aly,[a,*] Alaa A. Hassan,[a] Esraa M. Osman,[a] AbdElAziz A. Nayl,[b] Alan B. Brown[c] and Elshimaa M. N. Abdelhafez[d]

[a]*Chemistry Department, Faculty of Science, Minia University, El-Minia 61519, Egypt, e-mails:,asmaa.hamouda@mu.edu.eg, sara.ahmed@mu.edu.eg, ashrafaly63@yahoo.com(ashraf.shehata@mu.edu.eg), alaahassan2001@mu.edu.eg, esraamah33@gmail.com;* [b] *Department of Chemistry, College of Science, Jouf University, Sakaka 72341, Aljouf, Saudi Arabia, aanayel@ju.edu.sa;* [c] *Chemistry Department, Florida Institute of Technology, Melbourne, FL, United States;* [d]*Elshimaa M. N. Abdelhafez, Department of Medicinal Chemistry, Faculty of Pharmacy, Minia University, 61519 Minia, Egypt*
*Correspondence: ashrafaly63@yahoo.com, ashraf.shehata@mu.edu.eg

1. GENERAL INFORMATION

Melting points were determined on an electro thermal melting point apparatus (Stuart Scientific Co.) and were uncorrected. NMR spectra were measured on a Bruker AV-400 spectrometer (Bruker Bio Spin Corp., Billerica, MA, USA) (400 MHz for ^{1}H, 101 MHz for ^{13}C) at Florida Institute of Technology, USA. The ^{1}H and ^{13}C chemical shifts are given relative to internal standard TMS = 0. The description of signals includes: s = singlet, d = doublet, t = triplet, q = quartet, m = multiplet, dd = doublet of doublet and m = multiplet. The mass spectra were recorded on a Shimadzu GCMS-QGD-1000EX mass spectrometer at 70 eV (EI) at Cairo and Assiut University. The IR spectra were recorded in potassium bromide disks on Jasco FT/IR-450 Plus infra-red spectrophotometer. Elemental analyses were carried out at the Microanalytical Center, Cairo University, Egypt. TLC was performed on analytical Merck 9385 silica aluminum sheets (Kieselgel 60) with Pf254 indicator; TLC's were viewed at δmax = 254 nm.

2. COPIES OF NMR SPECTRA

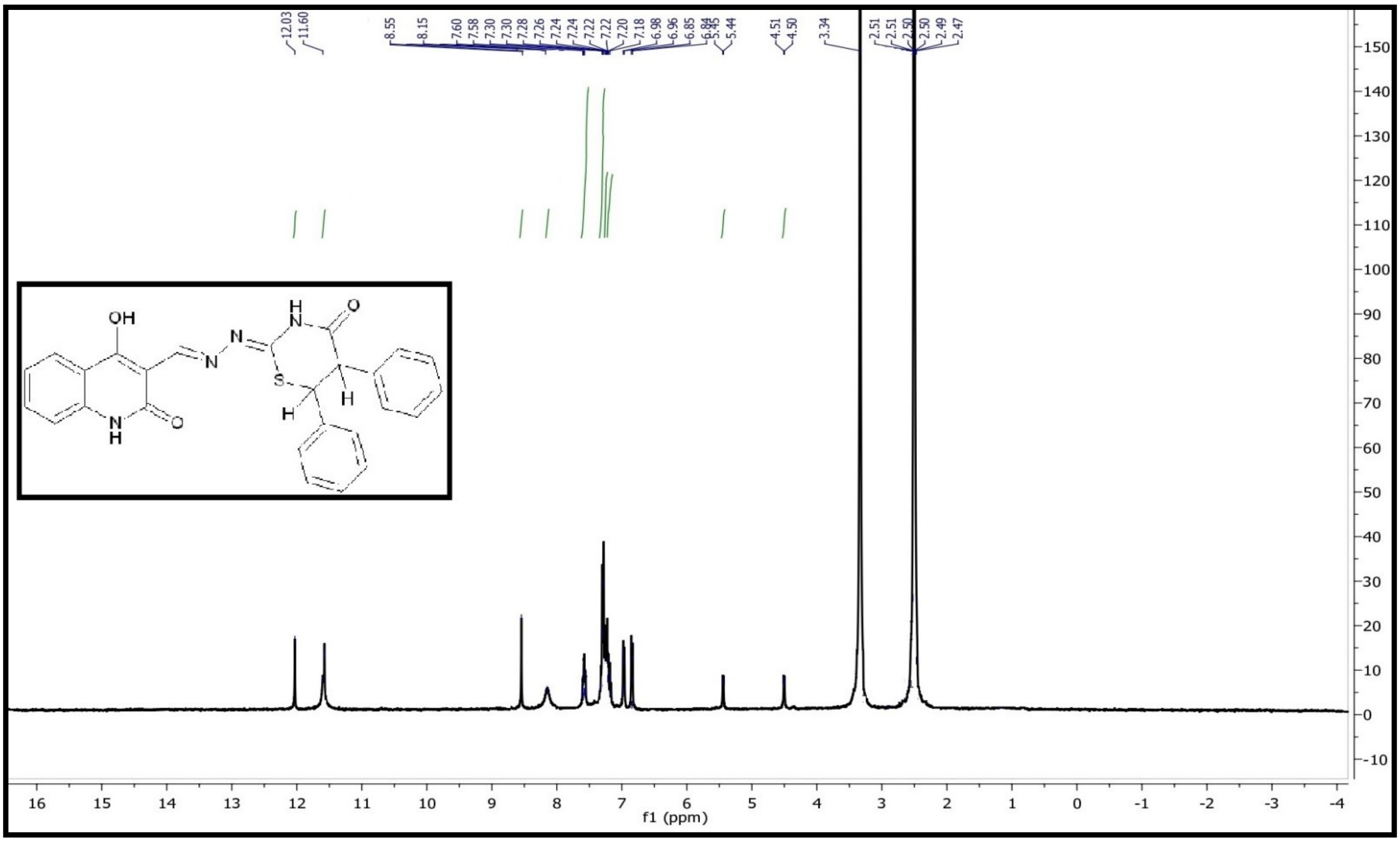

SI figure S1: ^{1}H-NMR spectrum of **7a**

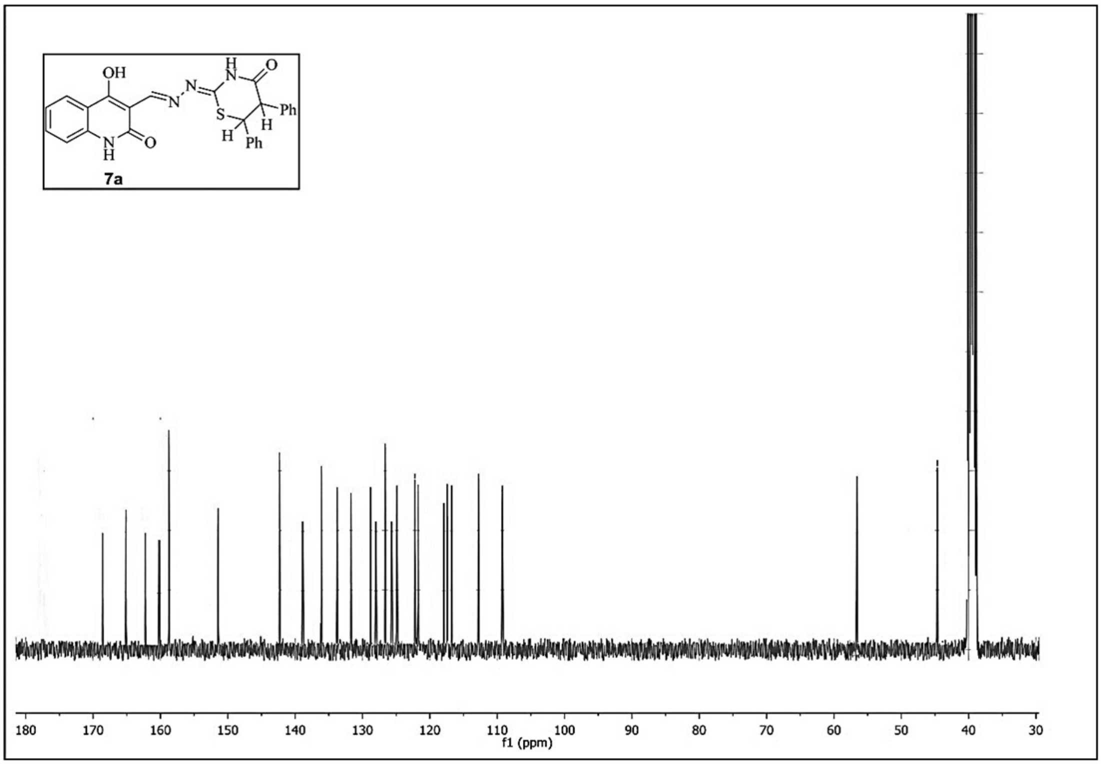

SI figure S2: ^{13}C-NMR spectrum of **7a**

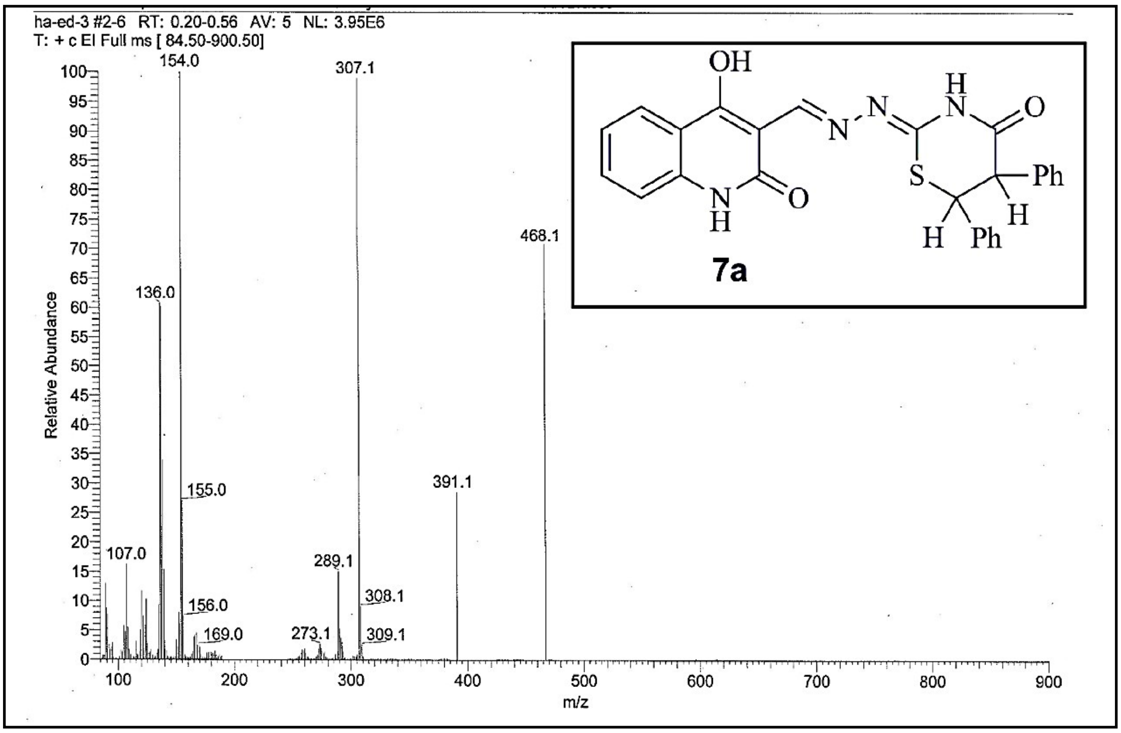

SI figure S3: Mass spectroscopy of **7a**

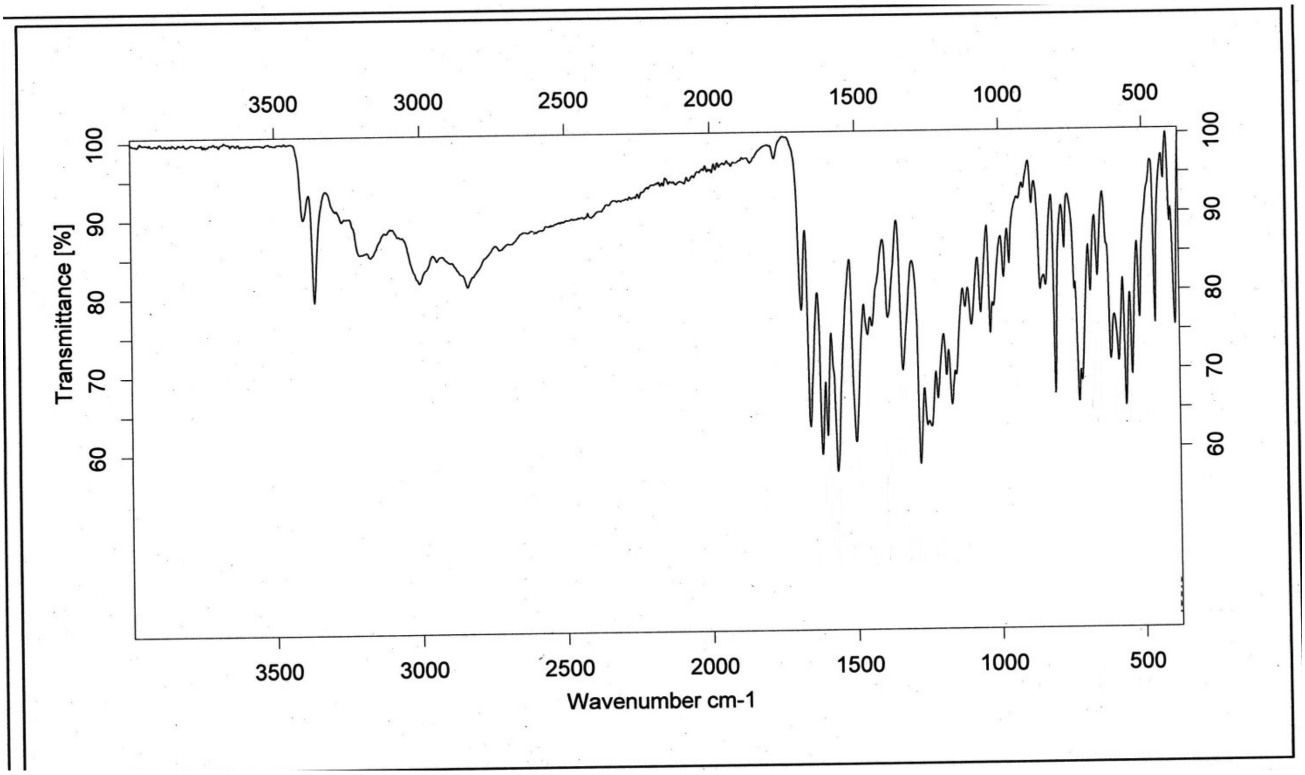

SI figure S4: IR spectra of **7a**

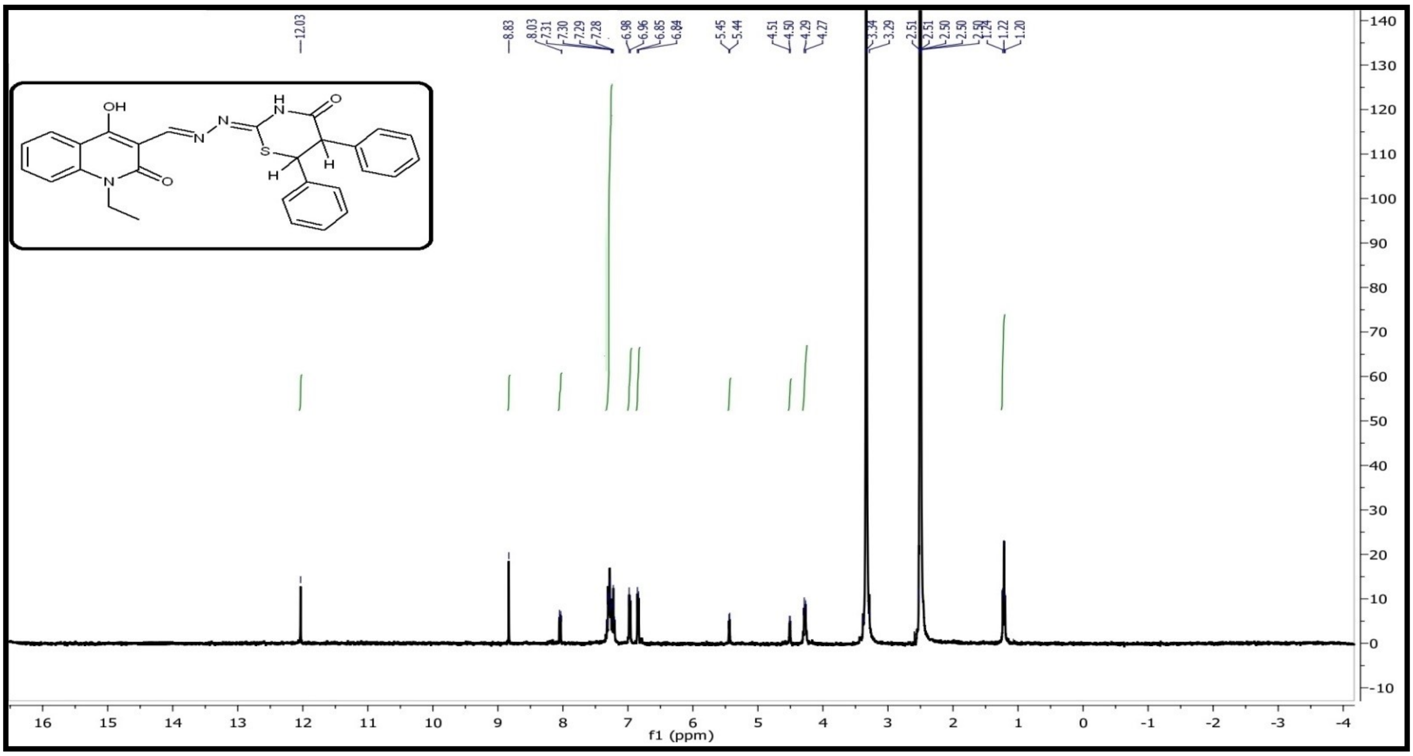

SI figure S5: ^{1}H-NMR spectrum of **7b**

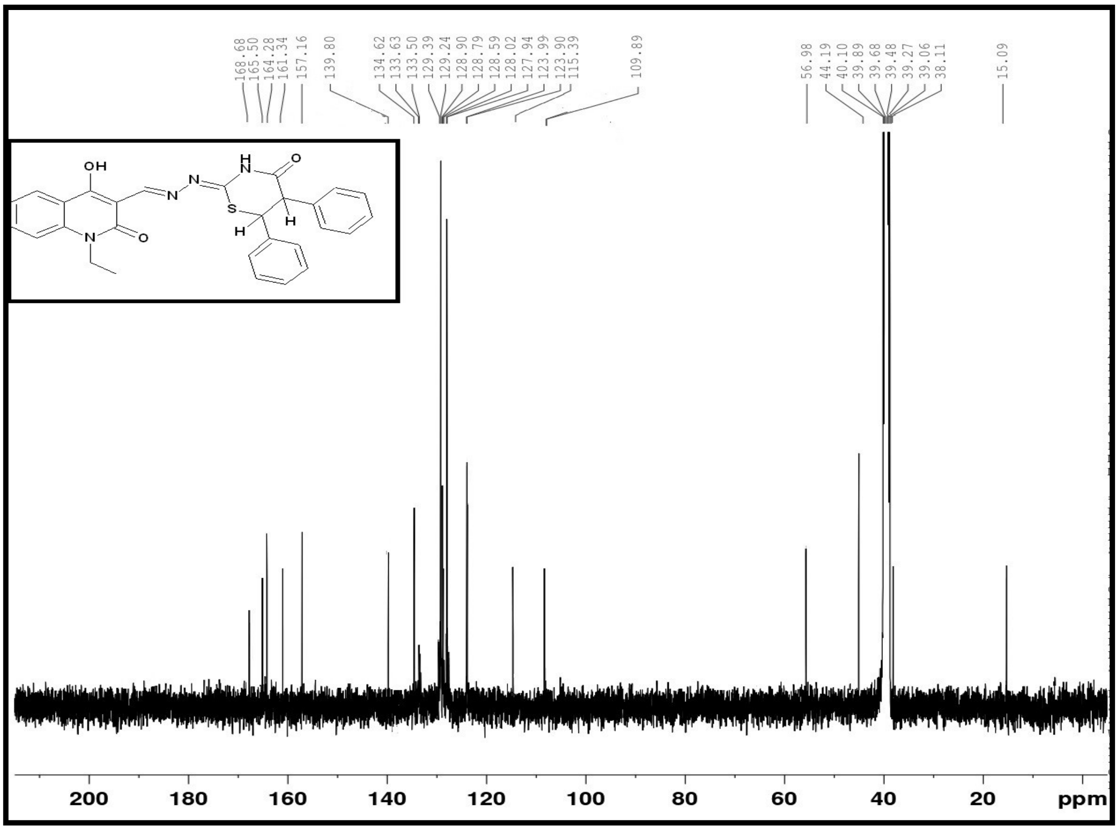

SI figure S6: ^{13}C-NMR spectrum of **7b**

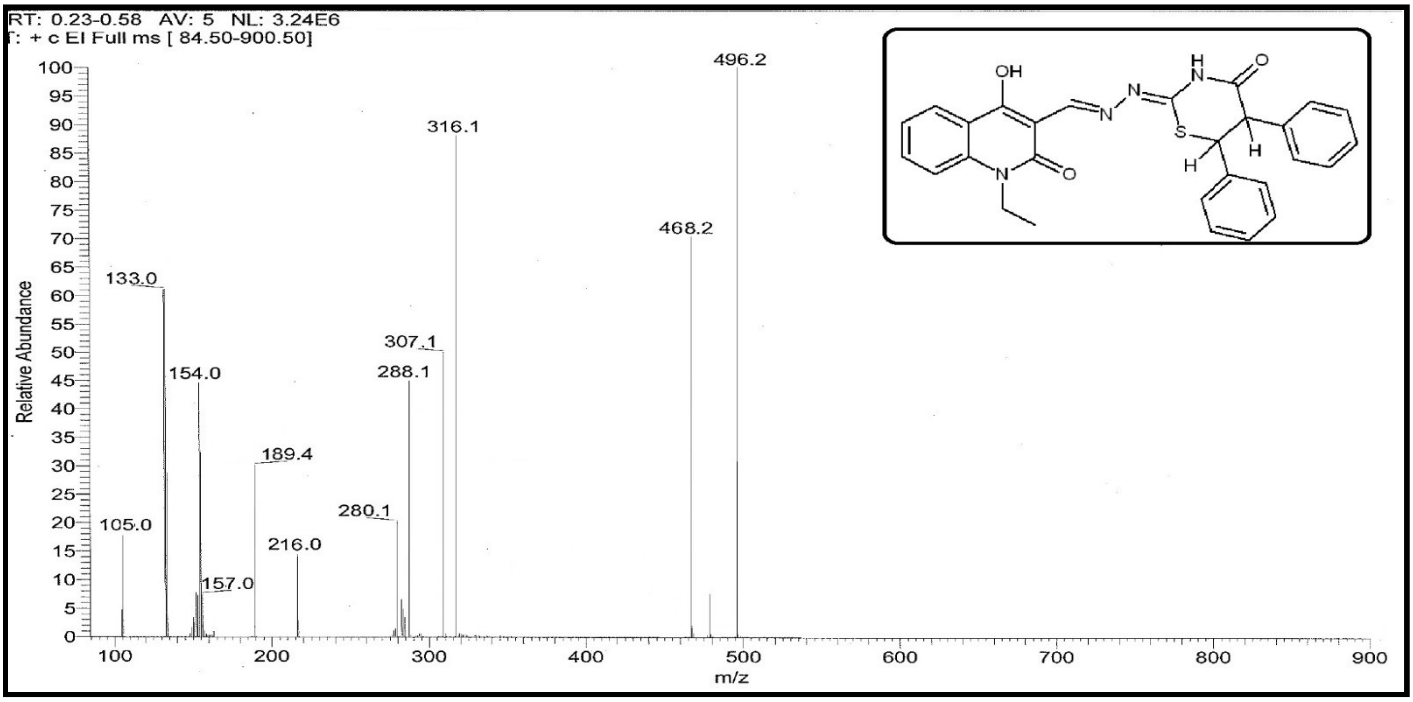

SI figure S7: Mass spectroscopy of **7b**

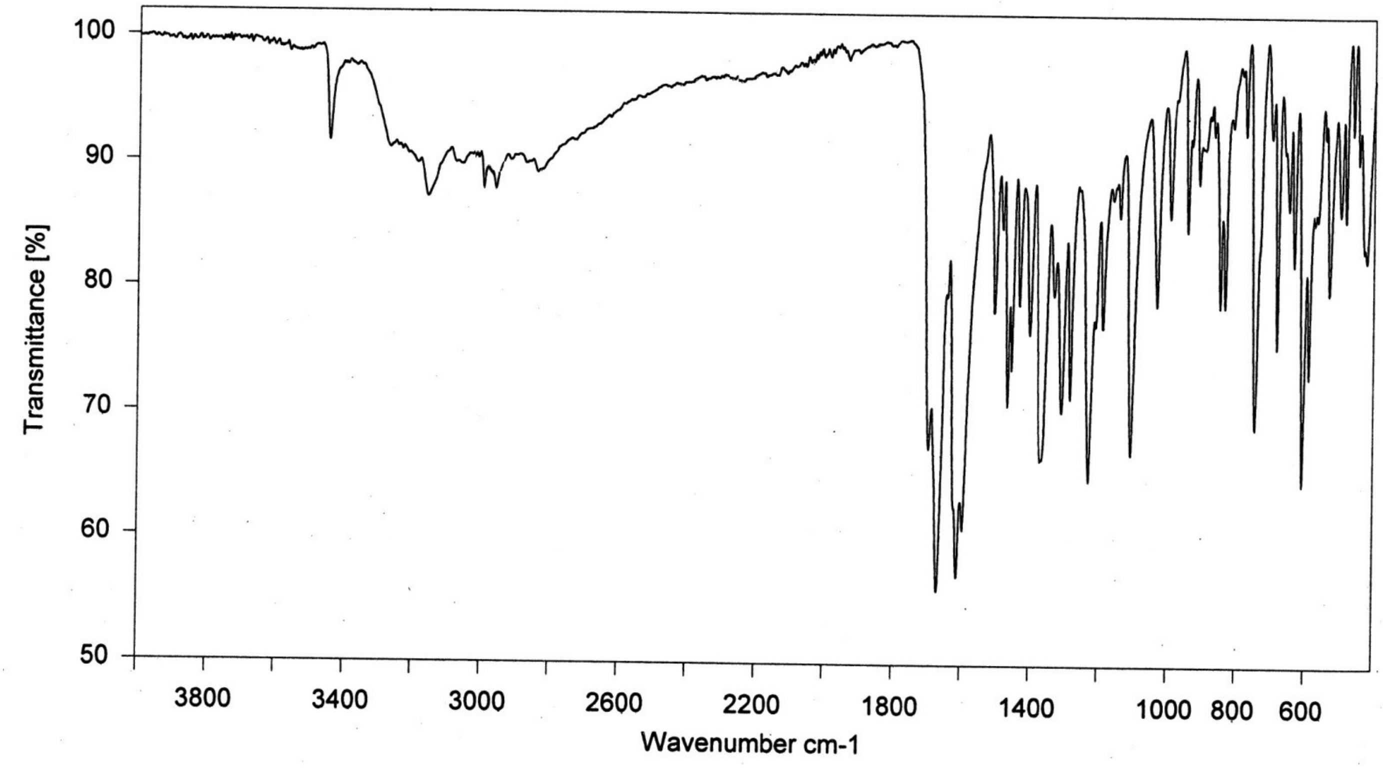

SI figure S8: IR spectra of **7b**

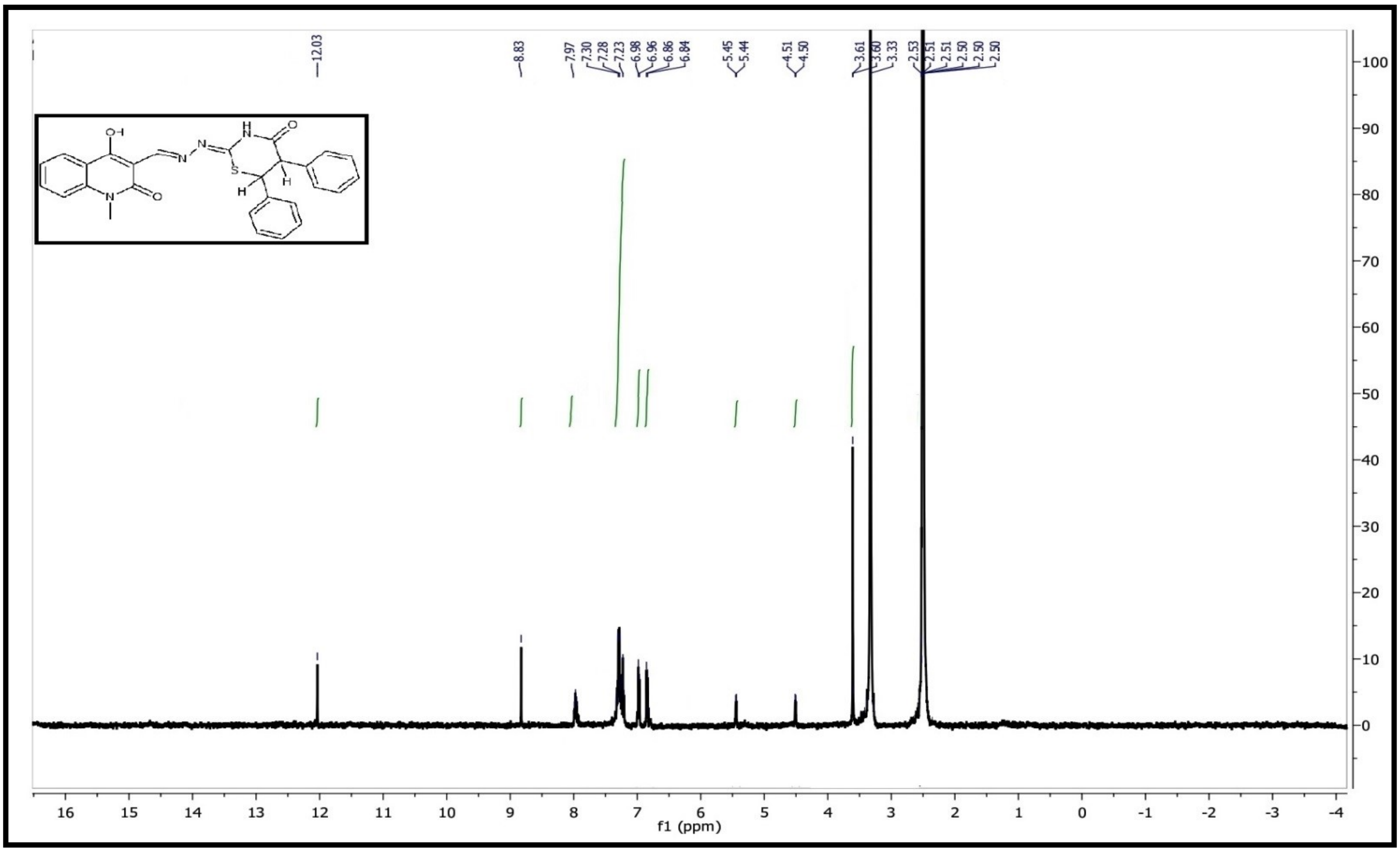

SI figure S9: ^{1}H-NMR spectrum of **7c**

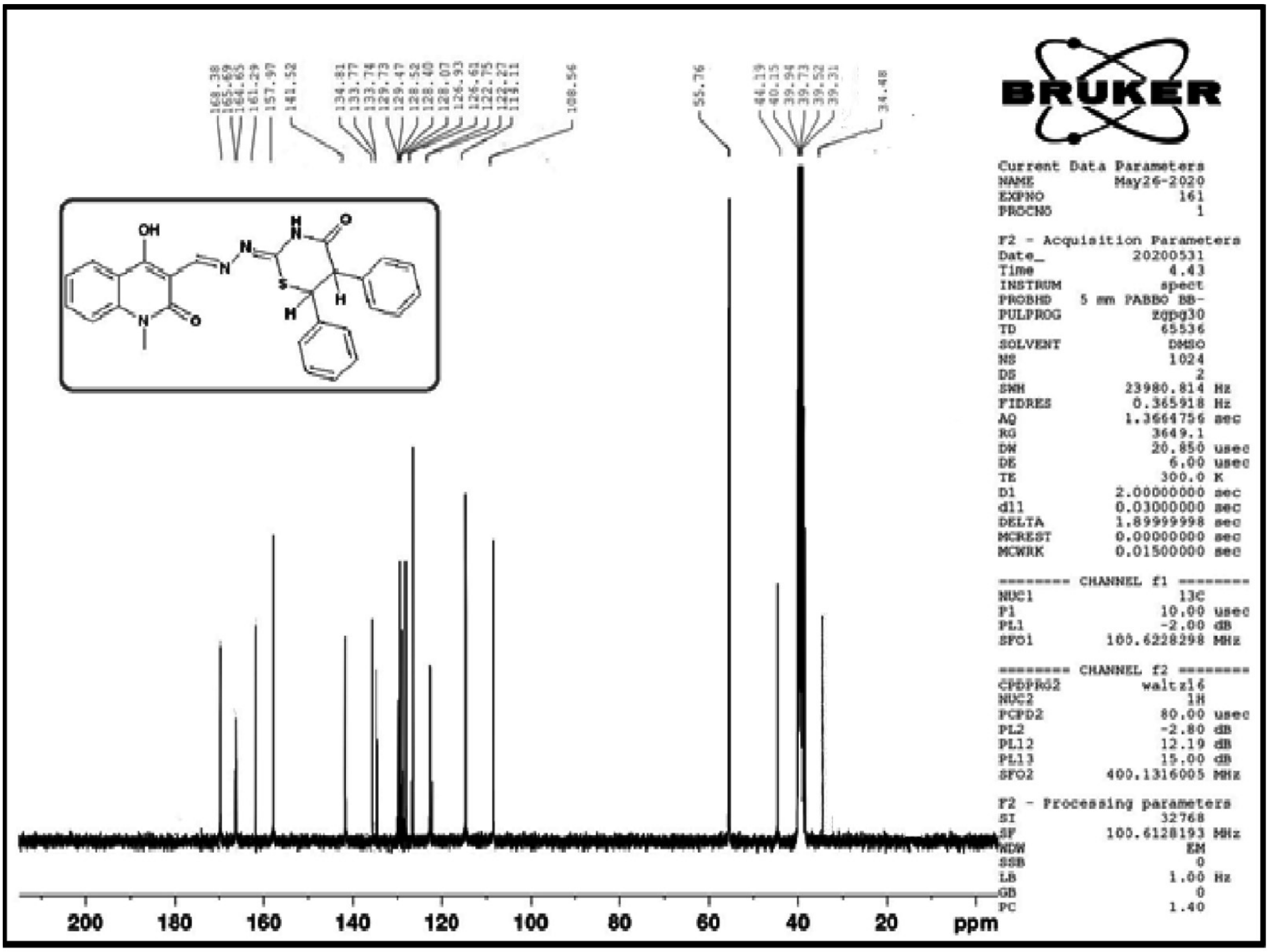

SI figure S10: ^{13}C-NMR spectrum of **7c**

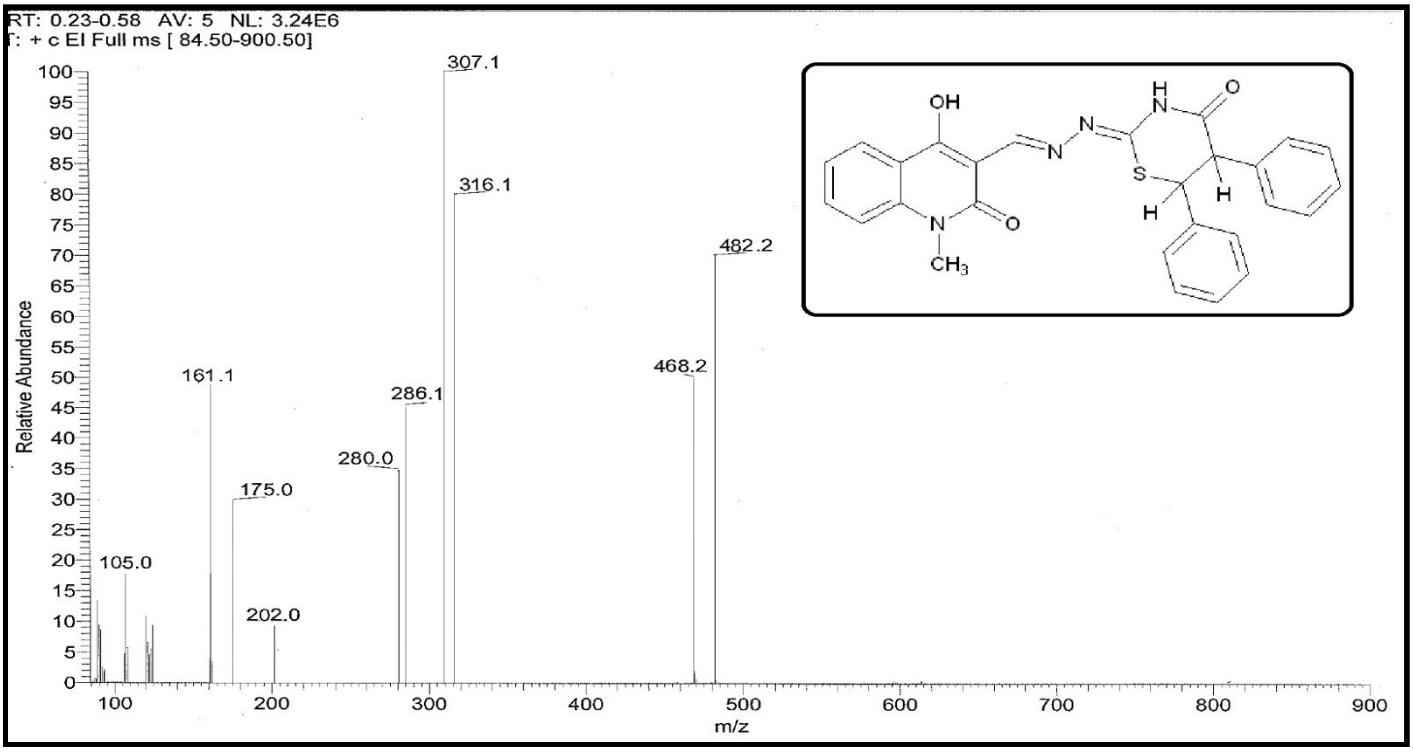

SI figure S11: Mass spectroscopy of **7c**

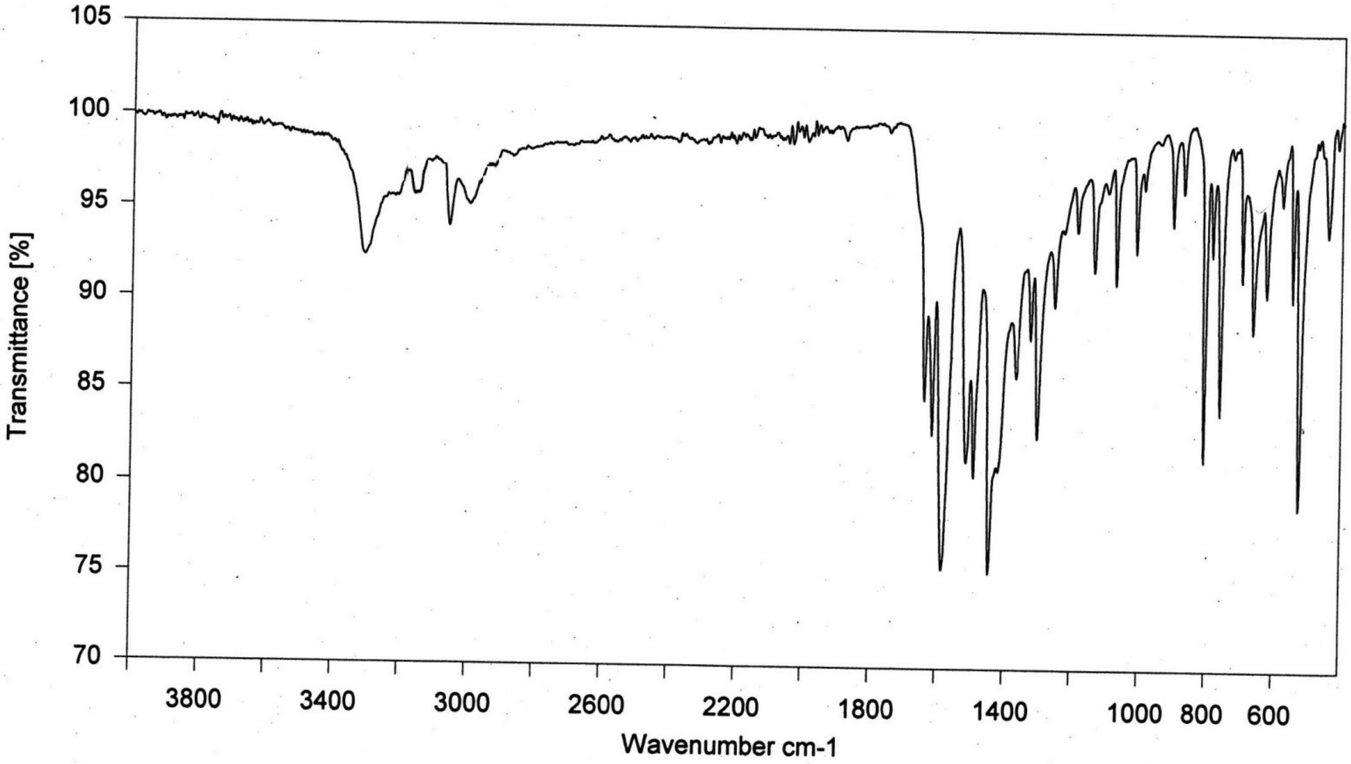

SI figure S12: IR spectra of **7c**

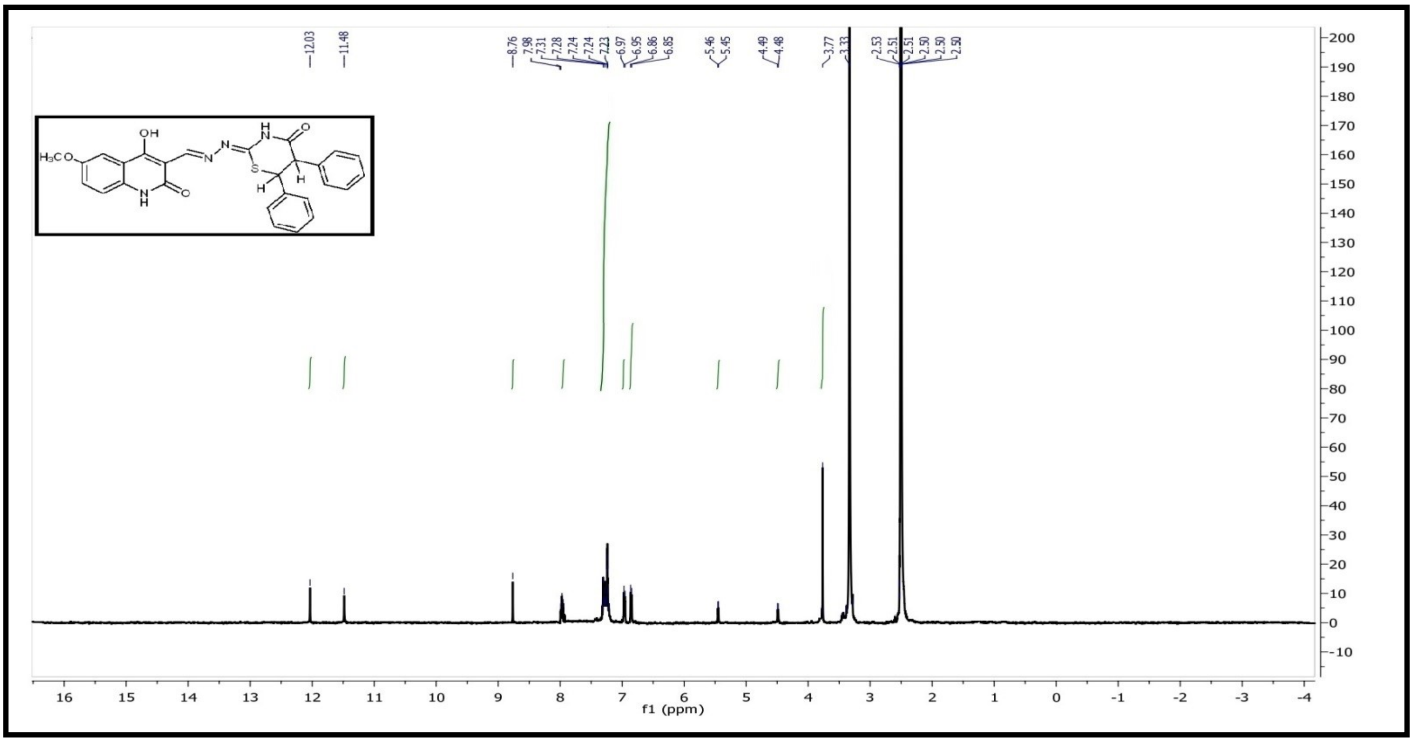

SI figure S13: ^{1}H-NMR spectrum of **7d**

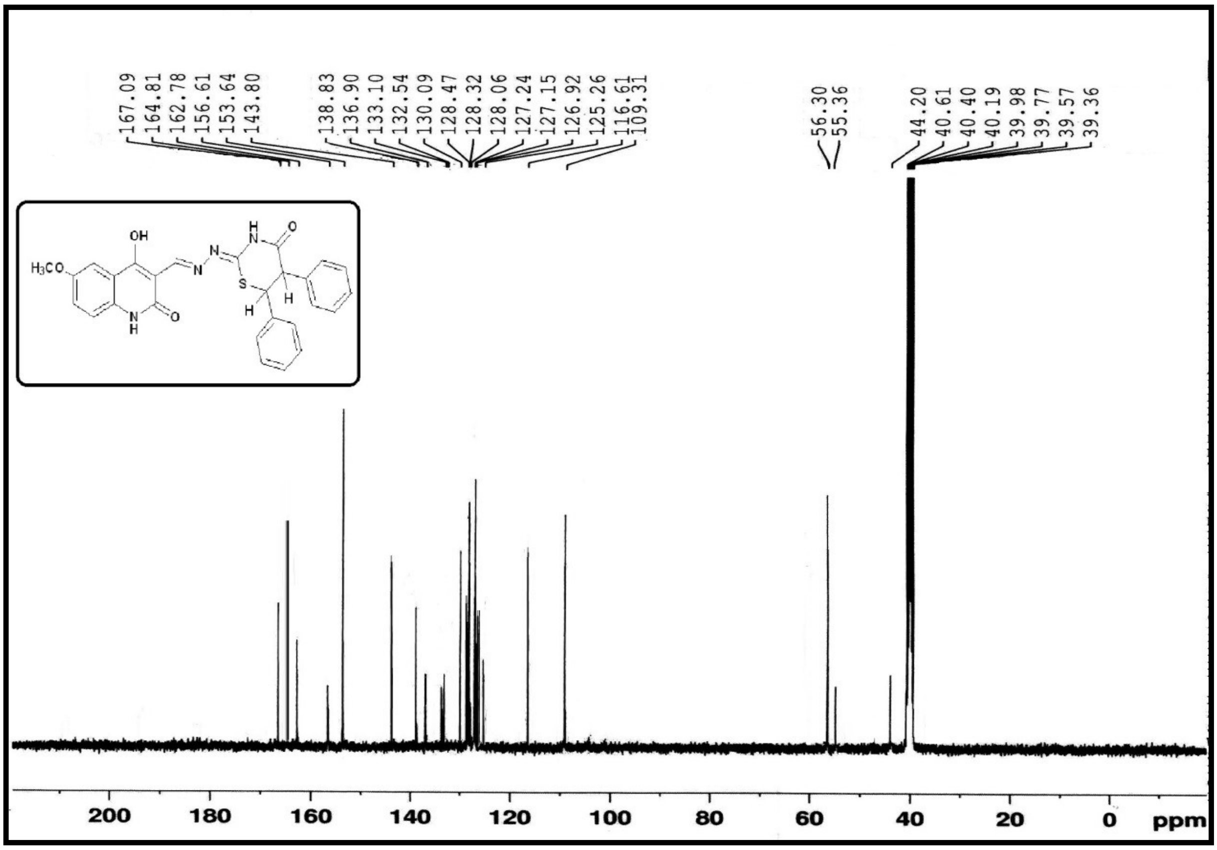

SI figure S14: ^{13}C-NMR spectrum of **7d**

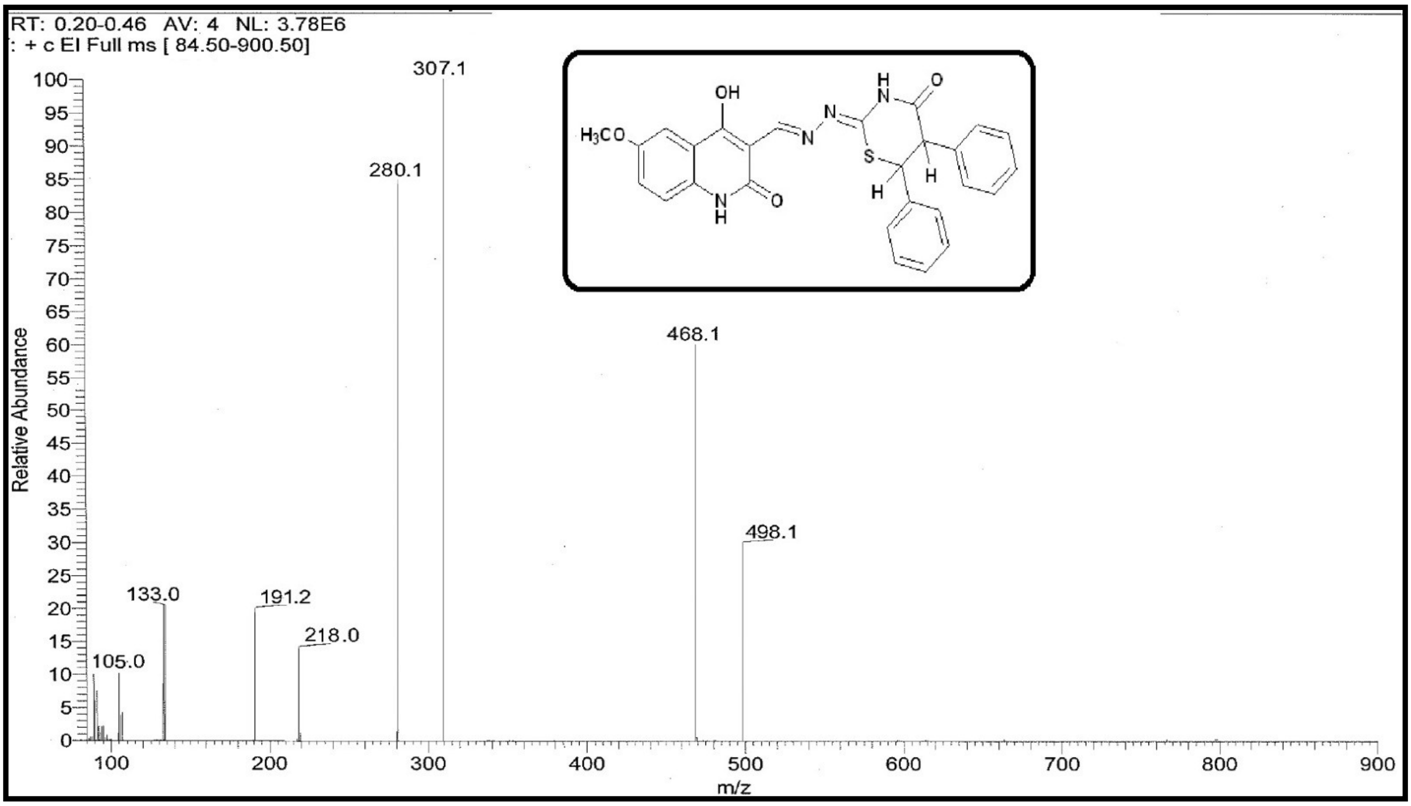

SI figure S15: Mass spectroscopy of **7d**

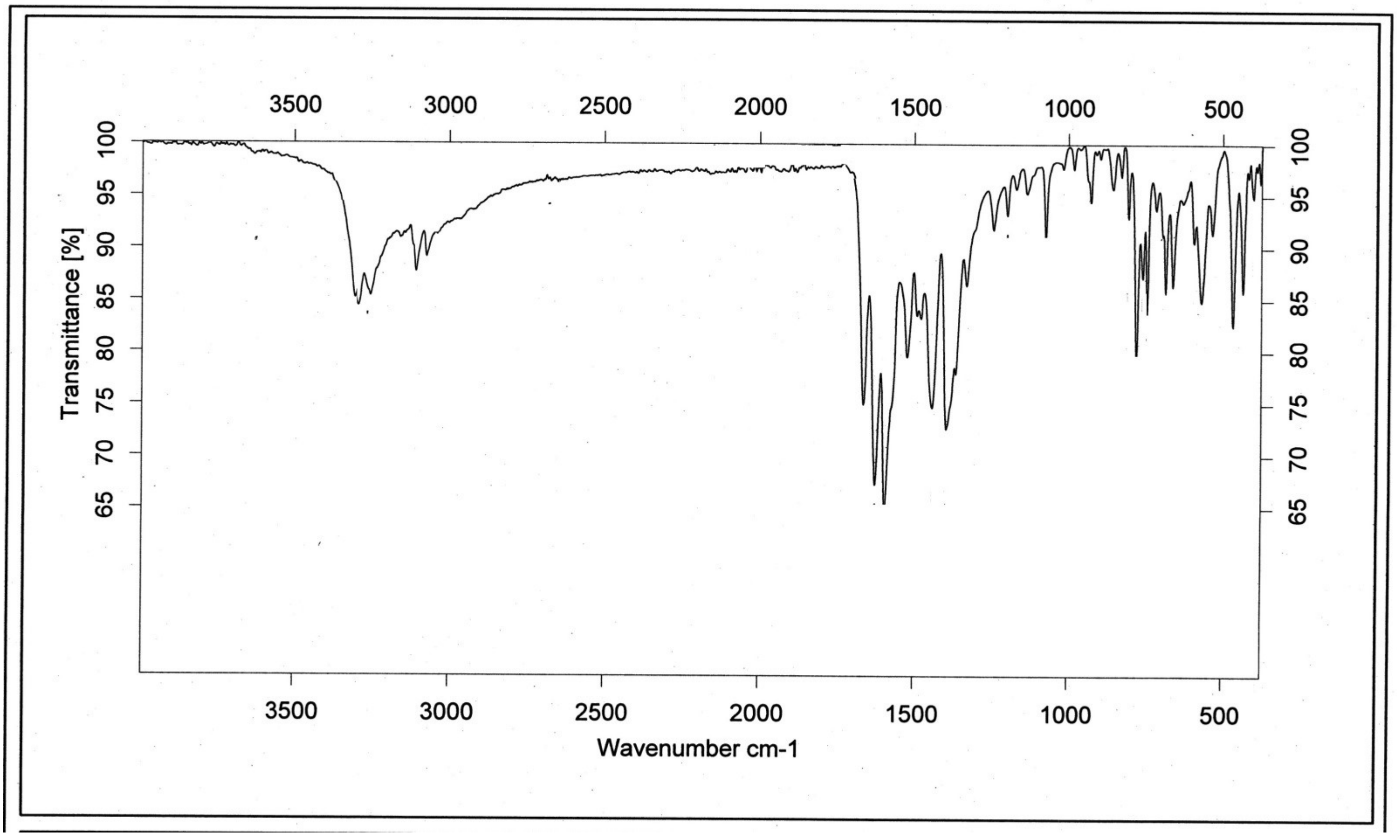

SI figure S16: IR spectra of **7d**

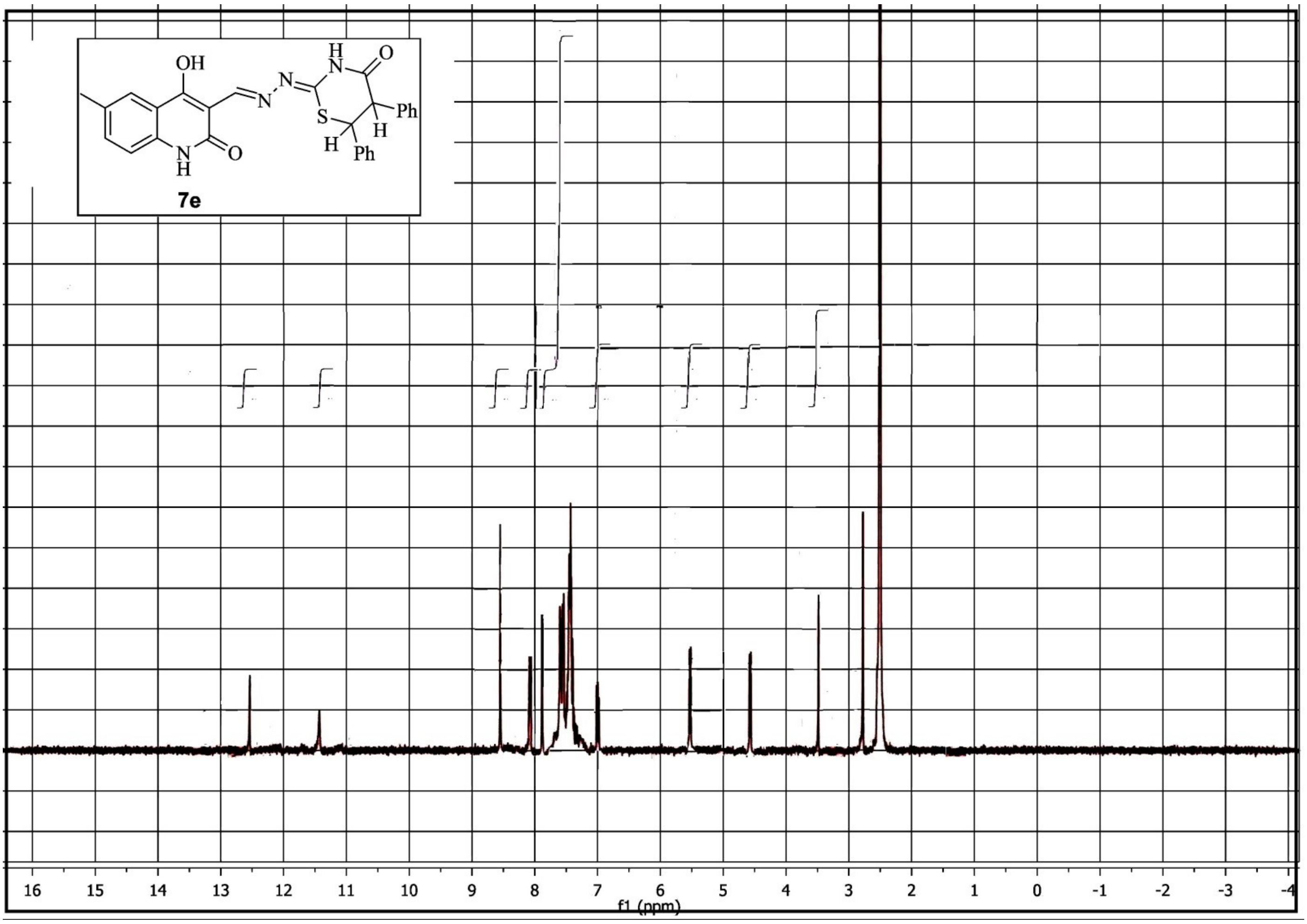

SI figure S17: ^{1}H-NMR spectrum of **7e**

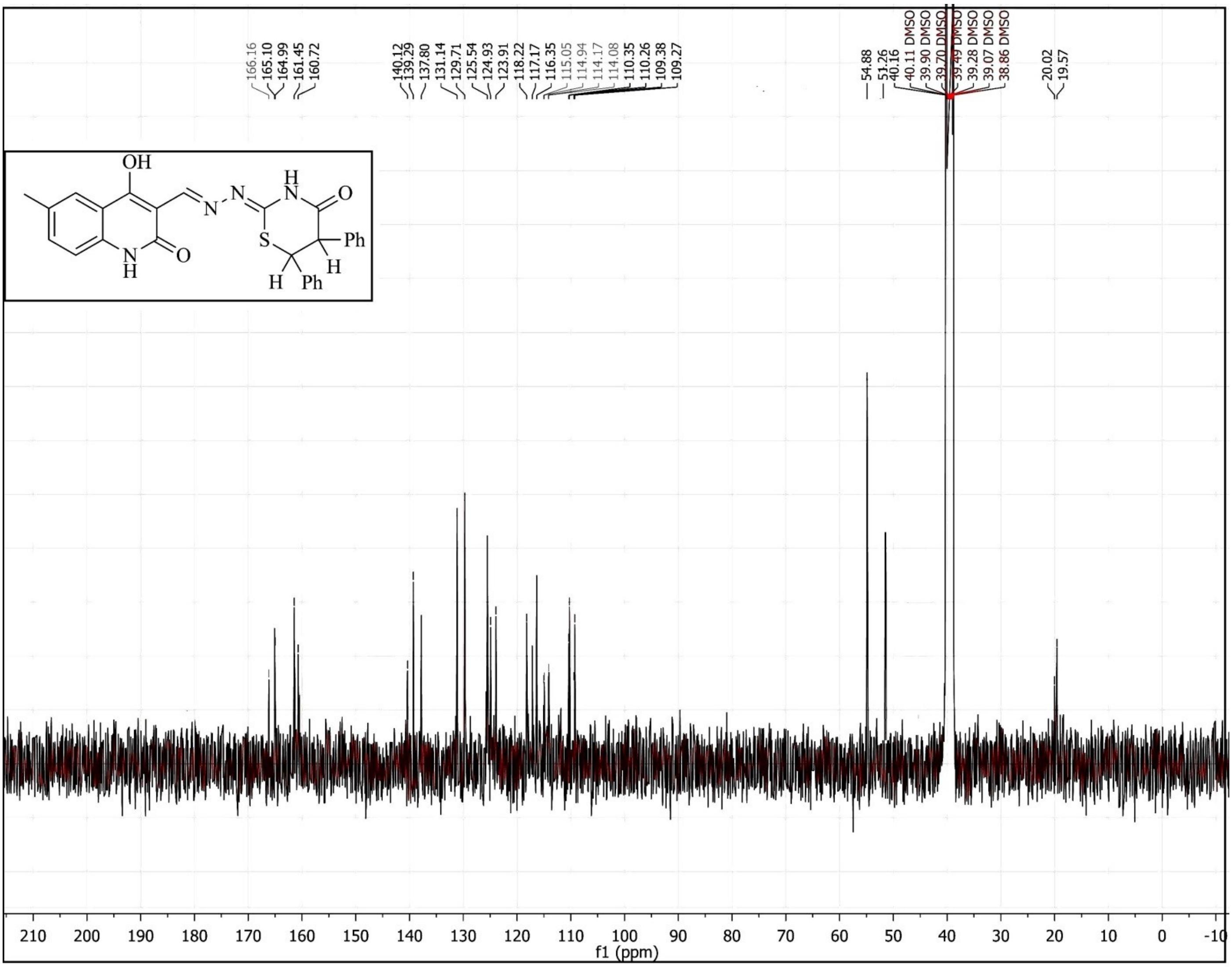

SI figure S18: ^{13}C-NMR spectrum of **7e**

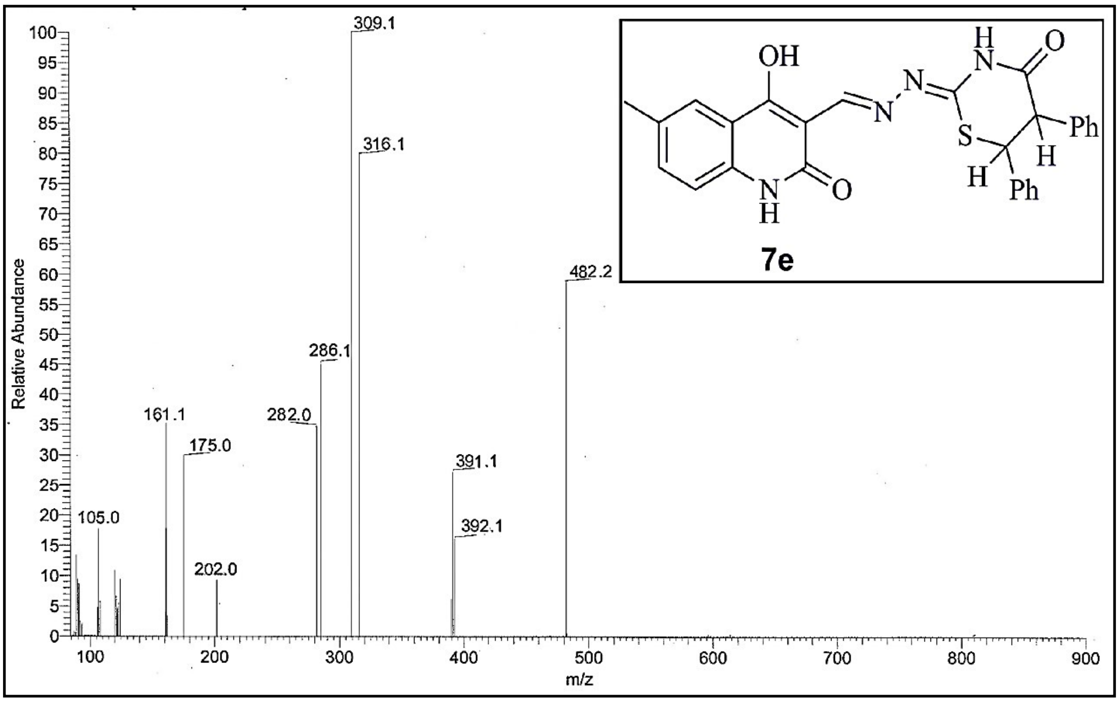

SI figure S19: Mass spectroscopy of **7e**

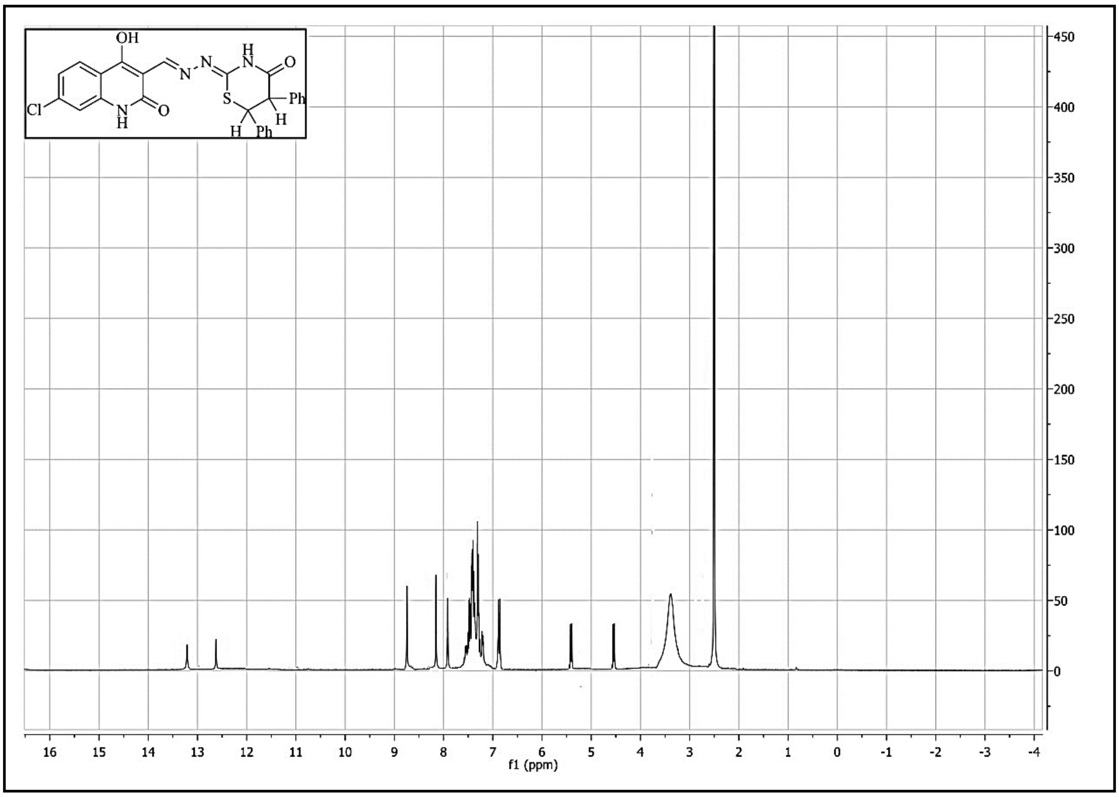

SI figure S20: ^{1}H-NMR spectrum of **7f**

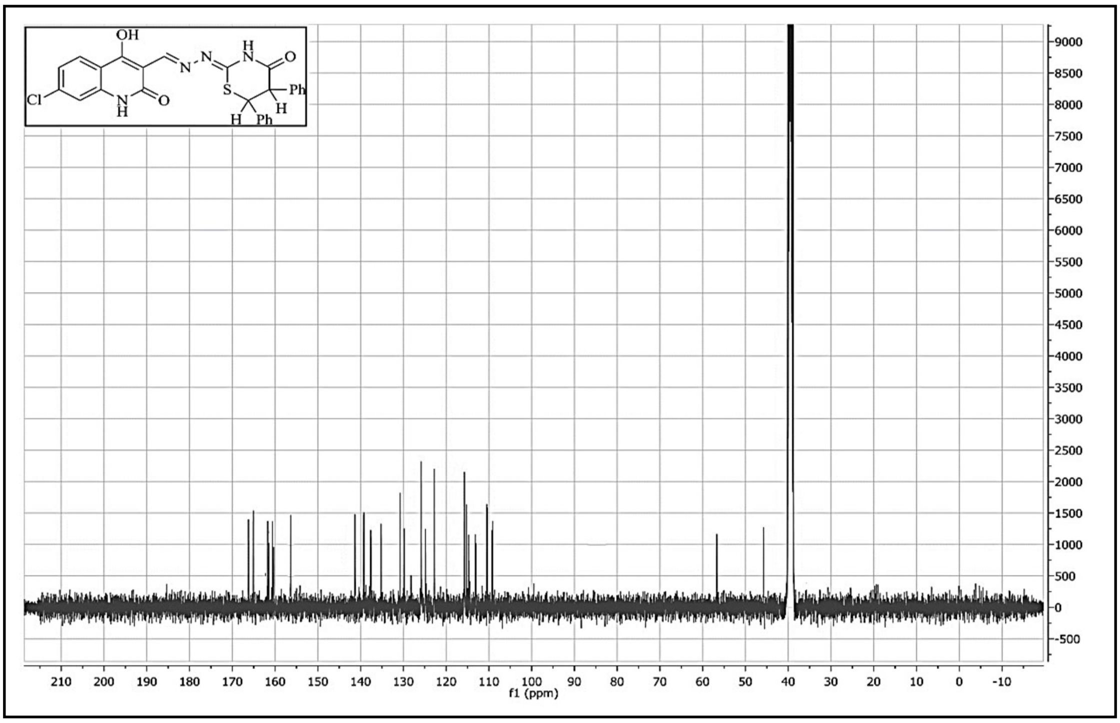

SI figure S21: ^{13}C-NMR spectrum of **7f**

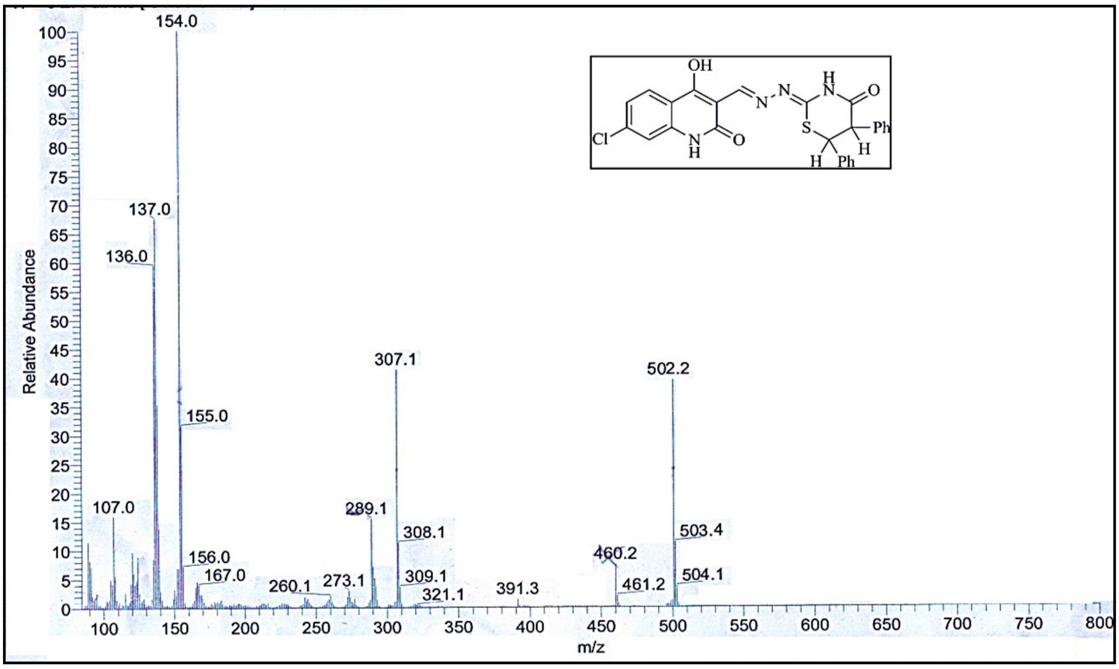

SI figure S22: Mass spectroscopy of **7f**

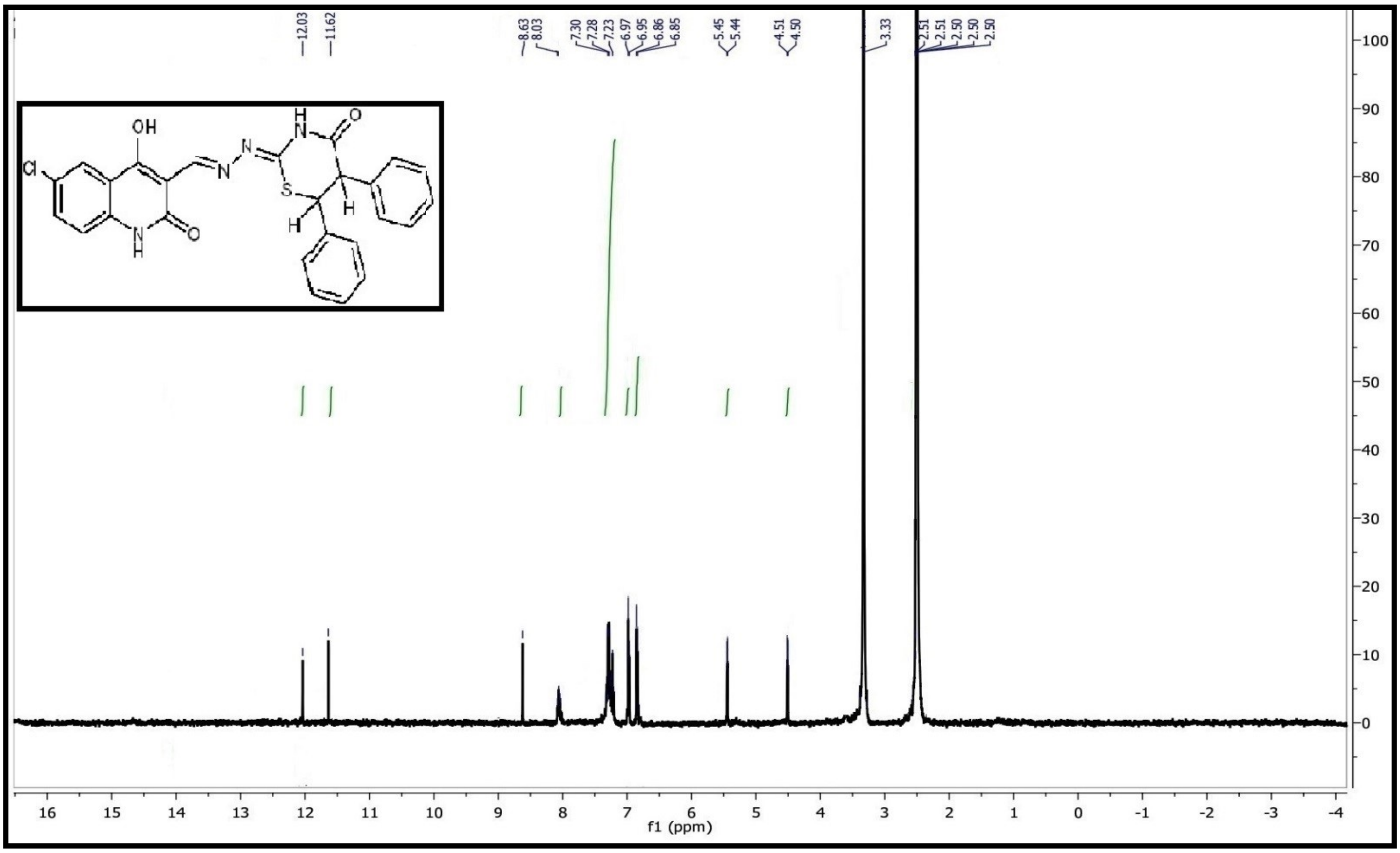

SI figure S23: ^{1}H-NMR spectrum of **7g**

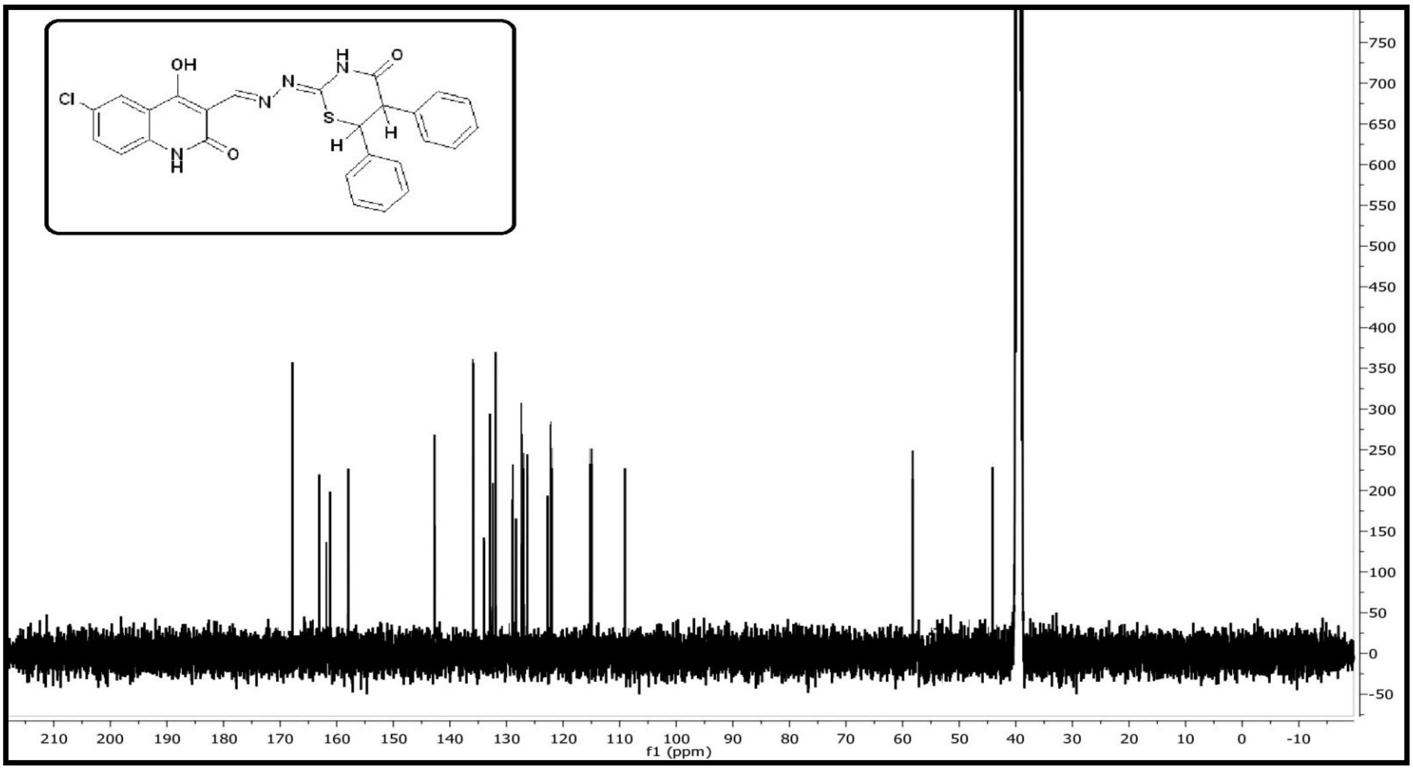

SI figure S24: ^{13}C-NMR spectrum of **7g**

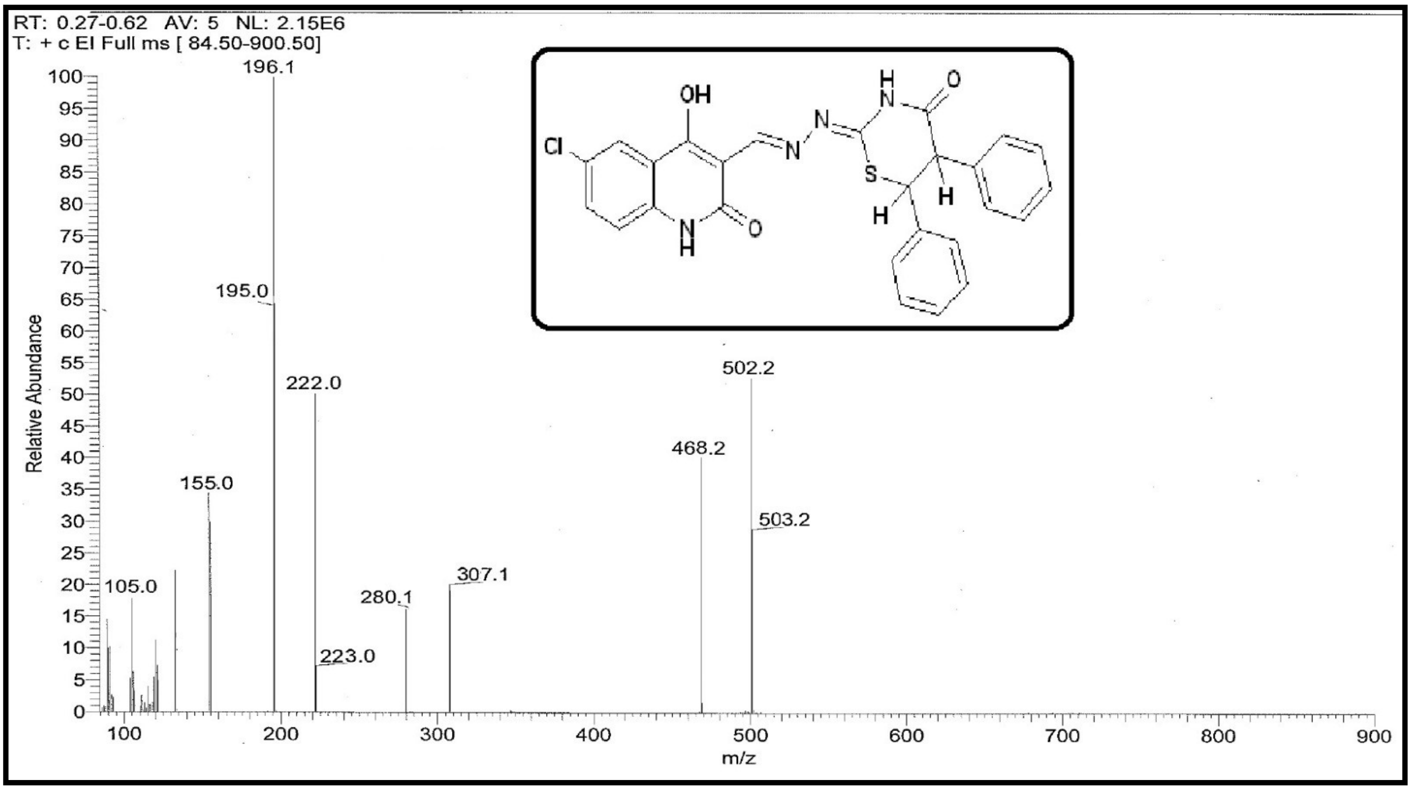

SI figure S25: Mass spectroscopy of **7g**

2. Screening of antibacterial activity

s	Strain	ATCC code	Batch no.
1	*Staph aureus*	6538	7T0218
2	*Staph aureus MRSA*	43300	
3	*E. coli*	8739	9e0218
4	*Pseudomonas aeruginosa*	9027	4p0218
5	*Salmonella*	14028	6s0218

Materials and Reagents

2. *Escherichia coli* (*E. coli*) (ATCC, catalog number: 25922)
3. Mueller Hinton broth (Sigma-Aldrich, catalog number: 70192)
4. Tigecycline (Wyeth, catalog number. 0220620-09-7)
5. NaCl (MDBio, catalog number: 101-1647-14-5)
6. KCl (Sigma-Aldrich, catalog number: P1147)
7. Na2HPO4 (J.T.Baker®, catalog number: 3828-01)
8. KH_2PO_4 (J.T.Baker®, catalog number: 4921-07)
9. HCl (J.T.Baker®, catalog number: 9535-03)
10. Tryptone (Pronadisa, catalog number: 1612)
11. Yeast extract (Pronadisa, catalog number: 1702)
12. Cation-adjusted Mueller-Hinton broth (CAMHB) (see Recipes)
13. PBS (1 L) (see Recipes)
14. Lysogeny broth (LB) (see Recipes)

Equipment

1. 50 ml polystyrene culture tubes (sterile)
2. Spectrophotometer to measure absorbance of cell culture (OD600)
3. 37 °C shaking and static incubators
4. Multichannel pipette (volume ranges 10 µl-1,000 µl)
5. 1.5 ml Eppendorf tube
6. A centrifuge machine
7. 1 ml cuvette

Procedure

A. Preparation of antibiotic stock solution and dilution range

1. Obtain antibiotic powder from the pharmaceutical company and make a note of the relevant information, including expiry date, potency, stability and solubility.

2. Prepare 1 ml 10 mg/ml tigecycline stock solution.

3. Choose a suitable range of antibiotic concentrations to be tested for *A. baumannii* if available. If the range is not available, maximal concentration 512 µg/ml and serial diluted concentrations with CAMHB solution are used. The lowest dilution concentration is depended on the possible minimal inhibition concentration. 0.125 µg/ml is the lowest possible dilution concentration.

4. To get different tested concentrations, solution of 10-time maximal concentration is prepared by dispensing the appropriate amount of stock solutions with micropipette and diluting with CAMHB solution.

For example, to get 1 ml 5,120 µg/ml solution, dispense 0.512 ml stock solution and dilute with 0.488 ml CAMHB solution.

B. Preparation of inoculum

1. Dissolve a single colony of *A. baumannii*, which is picked from a LB streak plate, in 3 ml LB broth and incubate overnight at 37 °C, 220 rpm.

2. Check OD600 (1 OD600= 109 CFU/ml) with a spectrophotometer.

3. Dilute the bacterial solution with LB broth to get 0.1 OD600 suspension and incubate at 37 °C, 220 rpm till mid-log phase (~2 h).

4. Put 1 ml mid-log phase bacterial solution in 1.5 ml Eppendorf tube, centrifuge at 6,000 rpm for five min, and wash with 1 ml PBS solution. Repeat the washing procedure twice.

5. Dissolve the bacterial pellet with 1 ml CAMHB solution.

6. Get 100 µl the above bacterial solution and mix it with 900 µl PBS, then check OD600with a spectrophotometer. The bacterial concentration can be deduced from the measured value x 10.

7. Adjust the bacterial concentration to 1 x 107 CFU/ml with CAMHB solution (1 OD600 ~109 CFU/ml).

C. Inoculation and incubation

1. Mix 50 µl adjusted *A. baumannii* bacterial solution (1x107 CFU/ml), 850 µl CAMHB and 100 µl solutions of 10-time serial tested antibiotic concentration. Use the *E. coli* ATCC25922 bacterial solution as a control.

2. Use 900 µl CAMHB and 100 µl solutions of 10-time serial tested antibiotic concentration for OD600 measurement comparison as a negative control.

3. Incubate at 37 °C, 220 rpm for 20-24 h.

D. Reading and interpretation

1. Check OD 600 with a spectrophotometer.

2. Read the MIC endpoint as the lowest concentration of antibiotic at which there is no visible growth of bacteria (no solution turbidity on naked eyes), and the difference of measured and background OD600 is less than 0.01.

Recipes

1. CAMHB

Dissolve 23 g Mueller Hinton broth in 0.9 L of distilled water
Adjust pH to 7.2 using HCl
Then fill up to 1,000 ml with distilled water
Sterilized by autoclaving at 121 °C for 15 min
And added
2 ml 10 g/L Ca^{2+} (8.36 g $MgCl_2.2H_2O$ in 100 ml ddH_2O)
1 ml 10 g/L Mg^{2+} (3.68 g $CaCl_2.6H_2O$ in 100 ml ddH_2O)
Stored at 4 °C

2. PBS (1 L)

8 g NaCl
0.2 g KCl
1.44 g Na_2HPO_4
0.24 g KH_2PO_4
Dissolve in 900 ml ddH_2O
Adjust pH to 7.2 using HCl
Sterilized by autoclaving at 121 °C for 15 min

3. LB

10 g tryptone
5 g yeast extract
5 g NaCl
Fill to 1 L with ddH_2O
Sterilized by autoclaving at 121 °C for 15 min

Molecular docking study

All conformers were subjected to energy minimization, all the minimizations were performed with MOE until a RMSD gradient of 0.01 Kcal/mole and RMS (Root Mean Square) distance of 0.1 Å with MMFF94X force-field and the partial charges were automatically calculated.

The obtained database was then saved as Molecular Data Base (MDB) file to be used in the docking calculations.

Optimization of the target:

The X-ray crystallographic structure of the target caspase-3 enzyme (PDB: 3GJQ) obtained from Protein data bank. The compounds were docked on the active site the target enzyme.

The enzyme was prepared for docking studies by:

The co-crystallized ligand, Moxifloxacin was deleted.

Hydrogen atoms were added to the system with their standard geometry.

The atoms connection and type were checked for any errors with automatic

Correction.

Selection of the receptor and its atoms potential were fixed.

Docking of the target molecules to caspase-3 enzyme active site

Docking of the target compounds was done using MOE-Dock software. The following methodology was generally applied:

The enzyme active site file was loaded, and the Dock tool was initiated. The program specifications were adjusted to:

- Dummy atoms as the docking site.
- Triangle matcher as the placement methodology to be used.
- London dG as Scoring methodology to be used and was adjusted to its default values.

The MDB file of the ligand to be docked was loaded and Dock calculations were run automatically.

The obtained poses were studied and the poses showed best ligand-enzyme interactions were selected and stored for energy calculations.
